# Divergent patterns of healthy aging across human brain regions at single-cell resolution reveal links to neurodegenerative disease

**DOI:** 10.1101/2023.07.31.551097

**Published:** 2023-08-01

**Authors:** Megan F. Duffy, Jinhui Ding, Rebekah G. Langston, Syed I. Shah, Mike A. Nalls, Sonja W. Scholz, D. Thad Whitaker, Pavan K. Auluck, Stefano Marenco, J. Raphael Gibbs, Mark R. Cookson

**Affiliations:** 1 Cell Biology and Gene Expression Section, Laboratory of Neurogenetics, National Institute on Aging, National Institutes of Health, Bethesda, Maryland, USA 20892; 2 Computational Biology Group, Laboratory of Neurogenetics, National Institute on Aging, National Institutes of Health, Bethesda, Maryland, USA 20892; 3 Data Tecnica International LLC, Washington, DC, USA; 4 Center for Alzheimer’s and Related Dementias, National Institutes of Health, Bethesda, MD, USA; 5 Neurodegenerative Diseases Research Unit, National Institute of Neurological Disorders and Stroke, Bethesda, MD, USA; 6 Department of Neurology, Johns Hopkins University Medical Center, Baltimore, MD, USA; 7 Human Brain Collection Core, Division of Intramural Research, National Institute of Mental Health, NIH, Bethesda, MD, 20892, USA

**Keywords:** Aging, putamen, entorhinal cortex, middle temporal gyrus, subventricular zone, snRNAseq, neurodegeneration, Alzheimer’s, Parkinson’s, dementia

## Abstract

Age is a major common risk factor underlying neurodegenerative diseases, including Alzheimer’s disease, Parkinson’s disease, and amyotrophic lateral sclerosis. Previous studies reported that chronological age correlates with differential gene expression across different brain regions. However, prior datasets have not disambiguated whether expression associations with age are due to changes in cell numbers and/or gene expression per cell. In this study, we leveraged single nucleus RNA-sequencing (snRNAseq) to examine changes in cell proportions and transcriptomes in four different brain regions, each from 12 donors aged 20–30 years (young) or 60–85 years (old). We sampled 155,192 nuclei from two cortical regions (entorhinal cortex and middle temporal gyrus) and two subcortical regions (putamen and subventricular zone) relevant to neurodegenerative diseases or the proliferative niche. We found no changes in cellular composition of different brain regions with healthy aging. Surprisingly, we did find that each brain region has a distinct aging signature, with only minor overlap in differentially associated genes across regions. Moreover, each cell type shows distinct age-associated expression changes, including loss of protein synthesis genes in cortical inhibitory neurons, axonogenesis genes in excitatory neurons and oligodendrocyte precursor cells, enhanced gliosis markers in astrocytes and disease-associated markers in microglia, and genes critical for neuron-glia communication. Importantly, we find cell type-specific enrichments of age associations with genes nominated by Alzheimer’s disease and Parkinson’s disease genome-wide association studies (GWAS), such as apolipoprotein E (*APOE*), and leucine-rich repeat kinase 2 (*LRRK2*) in microglia that are independent of overall expression levels across cell types. We present this data as a new resource which highlights, first, region- and cell type-specific transcriptomic changes in healthy aging that may contribute to selective vulnerability and, second, provide context for testing GWAS-nominated disease risk genes in relevant subtypes and developing more targeted therapeutic strategies. The data is readily accessible without requirement for extensive computational support in a public website, https://brainexp-hykyffa56a-uc.a.run.app/

## Graphical Abstract

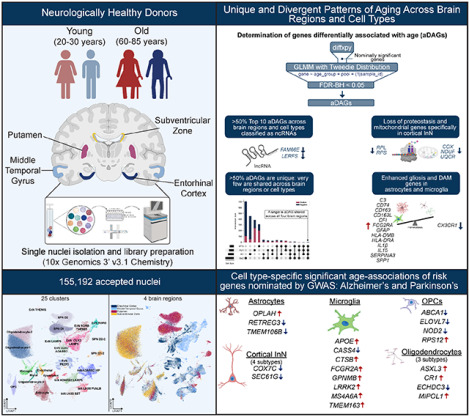

*Created using Biorender.com

## Introduction

Age is a common primary risk factor for multiple neurodegenerative diseases (NDDs), including Alzheimer’s disease and related dementias (ADRD), Parkinson’s (PD), and amyotrophic lateral sclerosis (ALS)^1^. For example, the incidence of AD approximately doubles in five-year increments between 60 and 90 years of age, and the prevalence of ADRD is expected to triple by 2050^2–4^. In this context, identifying the molecular events associated with healthy human brain aging becomes imperative in understanding predisposing conditions for neurodegeneration.

Prior studies used bulk transcriptomic and epigenomic analyses to understand the effects of aging on the human brain. The major themes emerging from these investigations include loss of synaptic gene expression and acquisition of inflammatory signaling networks^5^. Our previous RNA-sequencing (RNA-seq) analysis of the human dorsolateral prefrontal cortex identified networks of gene expression changes with age, including loss of neuronal genes^6^. For example, there was a strong decrease in *SST*, encoding the peptide neurotransmitter somatostatin, in a subset of GABAergic interneurons in the cortex.

Although this literature suggests robust changes in gene expression correlating with age, several aspects of the data remain difficult to interpret. For example, loss of neuronal markers could reflect a loss of neurons and/or changes in gene expression per neuron. Recent development of single-cell methods allows for the identification of cell types and the interrogation of gene expression in each cell type. Here, we applied snRNA-seq to disambiguate cell proportions and gene expression per cell in the context of healthy human brain aging across multiple brain regions. We compared the entorhinal cortex (EC), susceptible to AD pathology^7,8^, with another cortical region, the middle temporal gyrus (MTG)^9,10^. We also included the putamen (PUT), affected in Huntington’s disease (HD)^11,12^ and PD^13–15^, and the subventricular zone (SVZ) region permissive for neurogenesis during development, although whether this remains true in the adult human brain is still contested^16^. We find substantial numbers of brain region- and cell type-specific aging associations in gene expression during healthy aging. However, we also identify novel cell type- and brain region-specific differences in age-associated gene expression relevant to neurodegenerative diseases.

## Results

### Cell type composition of four brain regions

**Figure 1:**
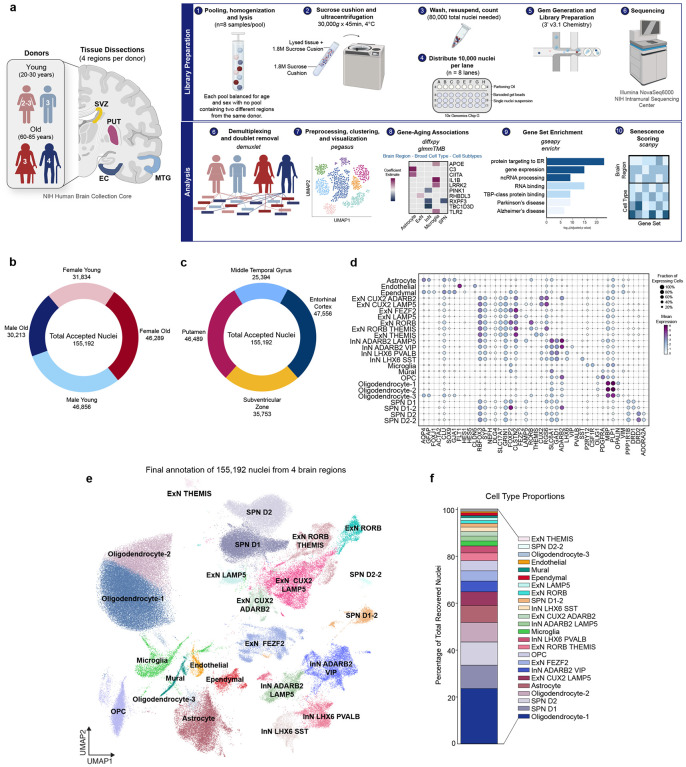
Experimental design and cell type composition of human entorhinal cortex, middle temporal gyrus, putamen, and subventricular zone **(a)** Schematic depicting experimental workflow for 10x Genomics snRNAseq library preparation and subsequent analysis of 4 brain regions from 12 individuals aged 20–30 (n=5–6) and 60–85 (n=7) balanced for sex (red, female; blue, male). See also: Methods. Created with BioRender.com. **(b)** Proportion of total accepted nuclei (155,192) split by sex and age **(c)** Proportion of total accepted nuclei (155,192) split by brain region **(d)** Dot plot of mean expression of multiple marker genes used to define each cell subtype. Size of circle denotes percentage of cells in a cluster expressing a gene and color represents strength of expression (purple, high expression; light blue, low expression). **(e)** UMAP of 155,192 annotated nuclei from all brain regions **(f)** Cell type proportions expressed as a percentage of total recovered nuclei **Abbreviations**: aDAG, aging-differentially associated gene; EC, entorhinal cortex; ExN, excitatory neuron; InN, inhibitory neuron; MTG, middle temporal gyrus; OPC, oligodendrocyte precursor cell; PUT, putamen; SPN, spiny projection neuron; SVZ, subventricular zone; UMAP, uniform manifold approximation and projection.

Our final dataset contained 155,192 nuclei from post-mortem EC (47,566 nuclei), MTG (25,394 nuclei), PUT (46,489 nuclei), and SVZ (35,753 nuclei) from six younger (20–30 years old) and six older (60–85 years old) adults following QC and preprocessing (see Methods; Fig. 1a–c; Supplemental Table 1). We projected nuclei from all donors and regions into a single UMAP and confirmed each cluster contained nuclei from all donors and both sexes (Supplemental Fig. 1a–e).First, we assigned Leiden clusters to general cellular categories (referred to as *broad type*) based on the expression of markers for neurons (*RBFOX3*, *SYP*, *GRIN1*, *GRIA2*; 51.32% of all nuclei), microglia (*CSF1R*, *AIF1*, *P2RY12*; 2.1%), astrocytes (*AQP4*; 7.27%), oligodendrocyte precursors (*PDGFRɑ*; 4.31%), ependymal cells (*FOXJ1*; 1.83%), and oligodendrocytes (*MBP*; Fig. 1d–f; Supplemental Fig. 1f–h). Following *broad type* classification, we examined the diversity of neuronal subtypes using a combination of canonical markers, differential expression, brain region origin, and previously published snRNAseq datasets^17,18^. We assigned each cell subtype according to its 1) broad type marker, 2) subtype marker, and 3) secondary cluster-specific marker (Fig. 1d).

Inhibitory neurons (InN) were broadly identified by expression of genes encoding glutamate decarboxylase 1 and 2 (*GAD1*, *GAD2)* and represented ~31.2% of recovered nuclei (Fig. 1d–f; Supplemental Fig. 1h). Cortical InNs were split by expression of either *ADARB2* or LIM Homeobox 6 (*LHX6*). *ADARB2*^+^ InNs were subdivided by expression of either *LAMP5* (2.07%) or vasoactive intestinal peptide (*VIP*, 4.92%). *LHX6*^+^ InN subtypes were further determined by expression of either somatostatin (*SST*, 1.89%) or parvalbumin (*PVALB*, 2.94%). Subcortical *GAD1*^+^ and *GAD2*^+^ InN (21.59%) consisted of putaminal spiny projection neurons (SPN), which were further divided by expression of dopamine receptor D1 (*DRD1*, 9.91%) or D2 (*DRD2*, 9.85%). We also identified subsets of each SPN subtype. SPN D1–2 neurons (1.54%) coexpressed *ADARB2*, while SPN D2–2 neurons (0.3%) coexpressed adenosine A2a receptor (*ADORA2A*; Fig. 1d).

Excitatory neurons (ExN), identified by expression of genes encoding vesicular glutamate transporters 1 and 2 (*SLC17A6* and *SLC17A7*; Fig. 1d, Supplemental Fig. 1h), represented 18.4% of recovered nuclei. Iterative clustering subdivided these into seven distinct clusters, which were primarily restricted to the EC and MTG (Supplemental Fig. 1c,h). ExN clusters were distinguished by differential expression of marker genes related to cortical layers as previously published^17^ (Fig. 1d–f).

Non-neuronal cells represented nearly half of all recovered nuclei (48.6%) and were found across all brain regions (Fig. 1f; Supplemental Fig. 1c–d). Oligodendrocytes separated into 3 clusters, all expressing myelin basic protein (*MBP*) and proteolipid protein 1 (*PLP1*). Although Oligodendrocyte-1 (23.51%) and Oligodendrocyte-2 (8.28%) clusters are directly adjacent in the UMAP space, Oligodendrocyte-1 nuclei displayed higher expression of oligodendrocytic myelin paranodal and inner loop protein (*OPALIN*), which is involved in oligodendrocyte differentiation. Therefore, Oligodendrocyte-1 may represent a less mature subtype than Oligodendrocyte-2. Oligodendrocyte-3 (0.5%) expressed canonical oligodendrocyte markers *MBP* and *PLP1* but were also positive for *GFAP* and *AQP4* and likely represent O2A progenitors (Fig. 1d–f).

### Diverging patterns of gene expression by brain region in absence of cell type proportion shifts

**Figure 2:**
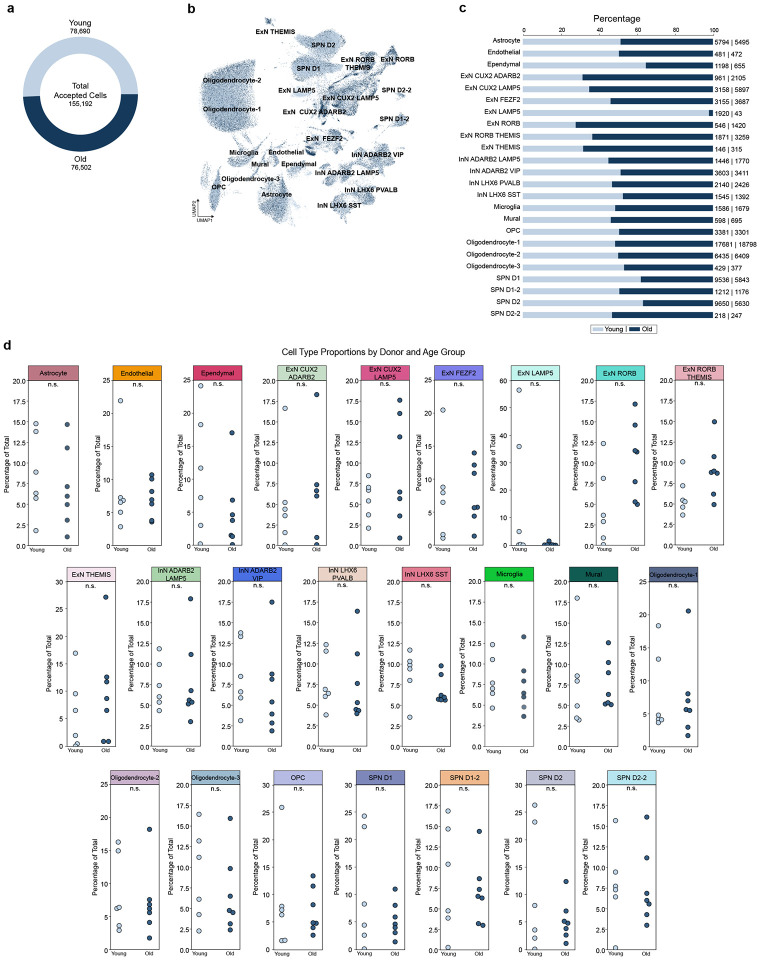
Cell type proportions are similar between young and old individuals **(a)** Total number of nuclei recovered by age group **(b)** UMAP of 24 cell type clusters colored by age group (young, light blue; old,dark blue) **(c)** Percentage of nuclei recovered for each cell type, split by age group (young, light blue; old, dark blue); proportion of recovered nuclei by cell type and age group raw numbers displayed as “young | old” **(d)** Cell type proportions split by age group, with each data point representing a single donor. Data expressed as percentage of total nuclei (young + old) recovered per cell type; n.s. = not significant; *p*>0.05, Welch’s t-test. **Abbreviations**: ExN, excitatory neuron; InN, inhibitory neuron; OPC, oligodendrocyte precursor cell; SPN, spiny projection neuron; UMAP, uniform manifold approximation and projection.

Due to limitations imposed by bulk RNAseq on interpreting if age-related expression patterns are due to cell type or gene expression changes, we examined whether cell proportions, particularly neuronal and oligodendrocyte subtypes, differed with age. The total number of nuclei recovered from young (78,690) and old (76,502) individuals was similar (Fig. 2a). There were no significant differences in cell type proportions with age for most cell types (Fig. 2b–d). The one exception was *LAMP5*^+^ExN, which were poorly recovered in the older age group and represented the most superficial layer of excitatory neurons^17^. However, even this difference was not statistically significant and is likely due to variability across individual donors (Fig. 2d). Additionally, the proportion of young versus old nuclei recovered by brain region did not significantly differ with age (Supplemental Fig. 2a–b). These results suggest that most aging-associated gene expression differences are not due to cellular proportions changes during aging

We next examined gene-aging associations at the level of brain region. For this analysis, we considered all nuclei within each of the four regions, irrespective of cell type. Using a two-step approach (see Methods), we found 2,071 genes exhibiting a significant differential association with age across the SVZ (767), EC (508), PUT (433), and MTG (363; Supplemental Fig. 2c; Supplemental Table 2). Most aging differentially associated genes (aDAGs) were unique to each brain region, with a single gene negatively associated with age (*AC240274.1*) intersecting all four regions. The SVZ and EC harbored more aDAGs with negative age associations than the PUT and MTG (641, 435, 157, 17 negative aDAGs, respectively), where >50% of significant genes were positively associated with aging. Of note, many aDAGs in each brain region were pseudogenes or long noncoding RNAs (lncRNAs). Of the top protein-coding genes most strongly associated with age at the brain region level, *HLA-DQA2*, *LILRA6*, *CD209*, *TNFSF14*, *TNFRSF10C*, *CCL3L1*, and *MUCL3* were notable in that they have established roles in the immune response (Supplemental Fig. 2d).

We next documented all gene-age associations at the level of *broad types* (Supplemental Table 2). Of 2,071 brain region aDAGs, 923 were also significant at the broad cell type level (Supplemental Table 3). At this resolution, cortical InN exhibited the most significant number of aDAGs, totaling 2,684 genes (Supplemental Table 2; Supplemental Fig. 2e). Many of the top aDAGs are lncRNAs or pseudogenes (Supplemental Fig. 2f). For example, the lncRNAs *FAM66E* and *LERFS* were decreased with age in all cell types except endothelial, ependymal, and mural cells. While a small subset of aDAGs was shared between ≥ 2 broad types, more than half of aDAGs were unique to each broad cell type.

### Negative association of protein translation and mitochondrial genes in cortical inhibitory neurons with age

**Figure 3:**
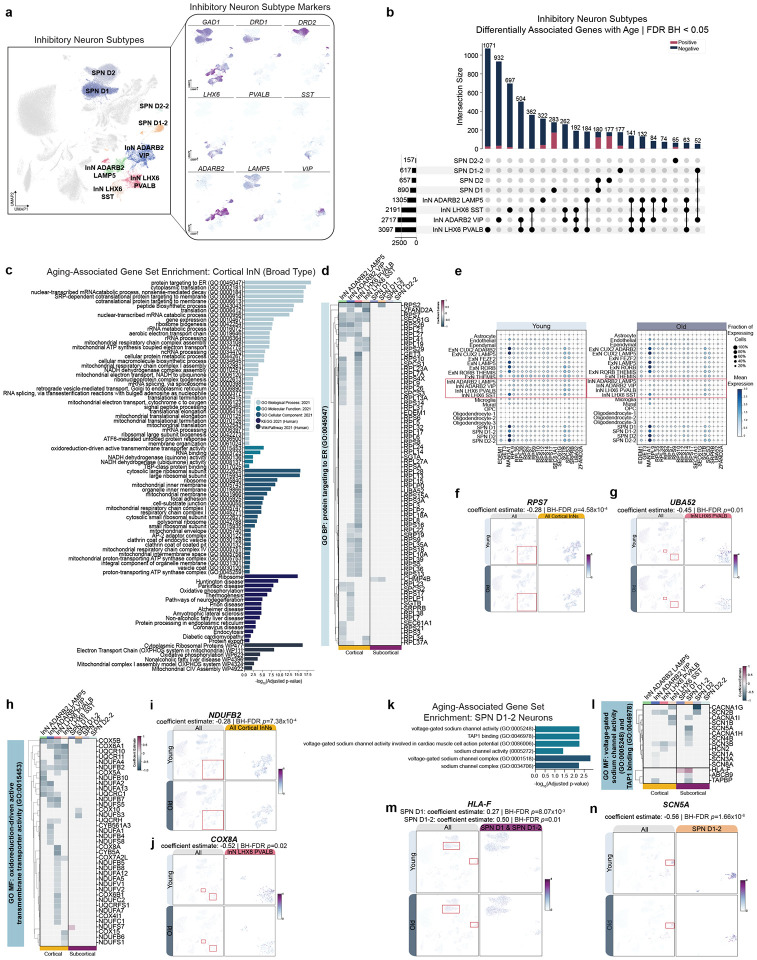
Diverging aging-associated changes in gene expression in cortical and subcortical spiny projection inhibitory neurons **(a)** UMAP of cortical and subcortical InN subtypes (left) and marker genes (right). **(b)** UpSet plot showing number of unique vs. shared aDAGs across cortical and subcortical InN subtypes, where rows on the x-axis correspond to [cell type] aDAG sets, columns correspond to intersection size between sets, and bar color corresponds to direction of association (dark blue, negative association with age; magenta, positive association with age). Lines between sets indicate shared aDAGs. Only intersection sizes of ≥50 are displayed. Cell type sets ordered by cardinality. **(c)** Significant GSE of cortical neuron aDAGs as a *broad type*. **(d)** Clustermap of significant aDAGs in “GO BP: protein targeting to ER (GO:0045047)” with strength of association enumerated as coefficient estimate (blue, negative association; magenta, positive association; grey, no significant association). Cell types ordered alphabetically. **(d)** Dot plot showing percentage of cells expressing (indicated by dot size) and mean expression (indicated by color) of a subset of “GO BP: protein targeting to ER (GO:0045047)” aDAGs, split by age group. Red asterisk indicates genes chosen to display in feature scatter plots. **(f)** Feature scatter showing expression *RPS7* and **(g)** *UBA52* across all clusters (left) and cluster[s] where the gene is significantly differentially associated with age (demarcated by red box and magnified on right). **(h)** Clustermap of significant aDAGs in “GO MF: oxidoreduction-driven active transmembrane transporter activity (GO:0015453).” Cell types ordered alphabetically. **(i)** Feature scatter showing expression of *NDUFB2 and* **(j)** *COX8A*. **(k)** GSEA of significant aDAGs in SPN D1–2 neurons. **(l)** Clustermap of significant aDAGs in: “GO MF: voltage-gated sodium channel activity (GO:0005248)” and “TAP1 binding (GO:0046978).” Cell types ordered alphabetically. **(m)** Feature scatter showing expression of *HLA-F* and **(n)** *SCN5A*. **Abbreviations**: aDAG, aging-differentially associated gene; BP, biological process; CC, cellular component; *COX8A*, cytochrome C oxidase subunit 8A; GO, gene ontology; GSE, gene set enrichment; *HLA-F*, major histocompatibility complex, Class I, F; InN, inhibitory neuron; KEGG, Kyoto Encyclopedia of Genes and Genomes; MF, molecular function; *NDUFB2*, NADH:ubiquinone oxidoreductase subunit B2; *RPS7*, ribosomal protein S7; *SCN5A*, sodium voltage-gated channel alpha subunit 5; SPN, spiny projection neuron; *TAP1*, transporter 1, ATP binding cassette subfamily B member; UMAP, uniform manifold approximation and projection.

We next examined all gene-age associations by cluster-specific cell types beginning with cortical InN and subcortical SPN broad classes, each composed of 4 cluster-specific cell types (Fig. 3a). We observed diverging directions of gene-aging associations between cortical InNs and subcortical inhibitory SPNs (Fig. 3b). Surprisingly, only 33 aDAGs were shared between the four cortical InN subtypes (Fig. 3b).

We next examined the top five aDAGs in each direction for cortical and subcortical InN subtypes. Similar to observations at the brain region and broad cell type levels, we noted an abundance of differentially associated lncRNAs, including *FAM66E* in all cortical InN subtypes and SPN D1–2 and D2 subtypes (Supplemental Fig. 3a). We next performed gene set enrichment analysis (GSEA) on both broad type and cluster-specific significant aDAG sets (Supplementary Information). Using the broad type cortical InN list of 2,864 aDAGs, we identified a large number of ontogeny enrichments clustered in themes of protein targeting and translation, gene expression, ncRNA processing, and mitochondrial electron transport (Fig. 3c; Supplementary Information). We then examined which genes within these GO term sets were differentially associated with age by plotting the coefficient estimate of enriched genes in different cell types (Fig. 3d, h, k). In cortical InNs, there was a striking negative association for ribosomal protein-coding genes in the GO term ‘protein targeting to ER (GO:0045047),’ particularly in the *LHX6*^+^*PVALB*^+^ InN cluster, but not in subcortical inhibitory SPNs (Fig. 3d). Several genes in this category were similarly negatively associated with age across multiple cortical InN subtypes, such as ribosomal protein S7 (*RPS7*) and SEC61 translocon subunit gamma (*SEC61G*; Fig. 3d,f). In contrast, many other genes in this category were negatively associated with age in a specific cell subtype, such as ubiquitin A-52 residue ribosomal protein fusion product 1 (*UBA52*) in *LHX6*^+^*PVALB*^+^ InNs only (Fig. 3d–e,g).

Given the strong negative association of these genes with age in cortical InNs, we next asked if these cells exhibited differential expression of these genes compared to other neuronal cell types in younger individuals. Surprisingly, young cortical InNs have lower expression of ribosomal protein-coding genes than excitatory neurons and subcortical inhibitory SPNs (Fig. 3e), showing that the decrease in protein synthesis genes with age in cortical InNs is not due to higher expression than other cell types.

In contrast to the abundance of ontology enrichments observed in cortical InNs at the level of broad cell type, only cluster-specific SPN D1–2 neurons exhibited enrichments (Supplementary Information). Notably, enrichment categories differed, and the enrichments’ strength was substantially lower than those observed in cortical InNs. Specifically, aDAGs in SPN D1–2 neurons fell under molecular function enrichment categories of voltage-gated sodium channel activity, calcium channel genes, and TAP1 binding activity (Fig. 3k–m). Notably, all SPN D1–2 aDAGs falling under enrichment categories were negatively associated with age, except for *HLA-F* (Fig. 3l–m).

Additionally, we examined the broad type- and cell-subtype gene enrichments for terms in the “Aging” and “Disease Perturbations” from GEO gene sets, subset by genes increased or decreased in expression with age or disease. Both cortical and subcortical InN-specific negative aDAGs significantly overlapped with genes previously shown in animals and humans to have decreased expression in aged cortical and hippocampal tissue. InN-specific negative aDAGs additionally overlapped with “Disease Perturbations (Down)” from both animal and human datasets including senescence, dystonia, ALS, and HD (Supplemental Fig. 3b–c; Supplementary Information).

### Excitatory neurons have categorically different and fewer aDAGs compared to inhibitory neurons

**Figure 4:**
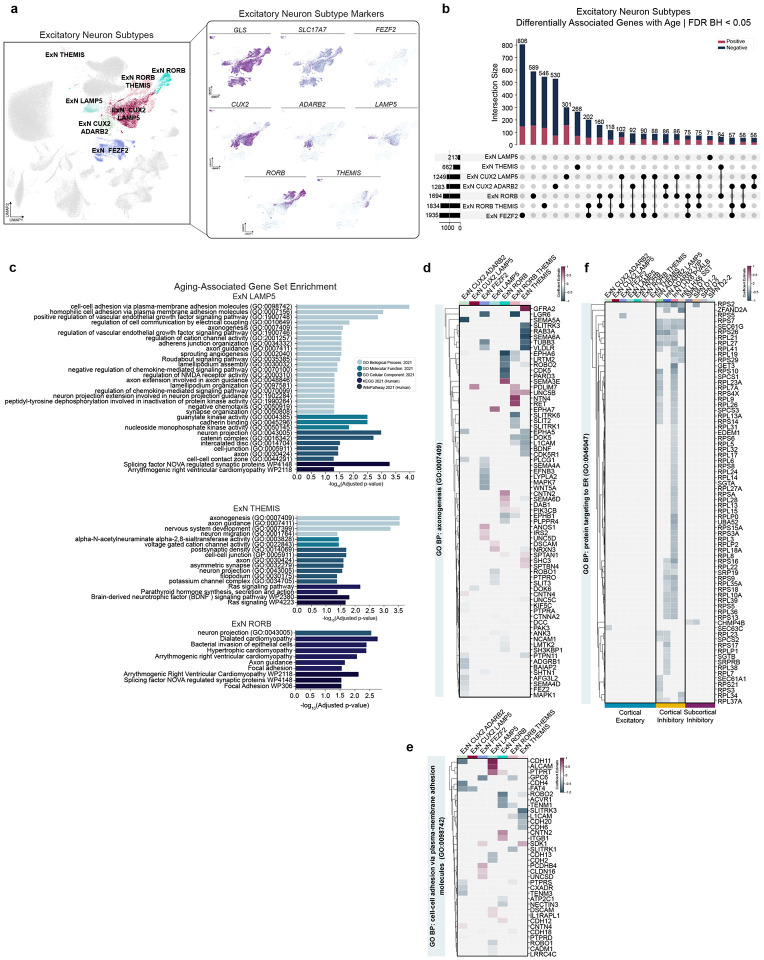
Aging-associated changes in gene expression between excitatory neuron subtypes differ in strength and GO categories from cortical inhibitory neurons **(a)** UMAP of cell type annotations (left) and feature plots depicting marker genes used to classify ExN subtypes (right). **(b)** UpSet plot depicting unique vs. shared aDAGs across ExN subtypes Only intersection sizes of ≥50 are displayed. Cell type sets ordered by cardinality. **(c)** Significant GSE in 3/7 ExN subtypes. **(d)** Clustermap of significant aDAGs in ExN under the term “GO BP: axonogenesis (GO:0007409).” Strength of association enumerated as coefficient estimate. Cell types ordered alphabetically. **(e)** Clustermap of significant aDAGs in ExN under the term “GO BP: cell-cell adhesion via plasma-membrane adhesion molecules (GO:0098742).” Cell types ordered alphabetically. **(f)** Clustermap comparing significant aDAGs under the category of “GO BP: protein targeting to ER (GO:0045047)” between cortical InN, cortical ExN, and subcortical InN. Cell types ordered alphabetically. **Abbreviations**: aDAG, aging-differentially associated gene; BP, biological process; CC, cellular component; ExN, excitatory neuron; GO, gene ontology; InN, inhibitory neuron; KEGG, Kyoto Encyclopedia of Genes and Genomes; MF, molecular function; SPN, spiny projection neuron; UMAP, uniform manifold approximation and projection.

We next examined ExNs, which had substantially fewer aDAGs (1,060) compared to InNs at the broad type level (Supplemental Table 2). ExN were clustered into seven subtypes (Fig. 4a). We observe the most aDAGs in *FEZF2*^+^ ExN representing layers 4–6 of the cortex (1250 total, 806 unique; Supplemental Table 2; Fig. 4b)^17^. Many top aDAGs were shared between ExN subtypes, including Rhomboid Like 3 which was previously documented to increase with aging in human frontal cortex^6,19^ (*RHBDL3*; Supplemental Fig. 4a–b). Interestingly, in the case of some related genes like calcium channel regulators *STAC* and *STAC2*, we observed association in opposing directions, sometimes within the same cell type cluster (Supplemental Fig. 4a, c–d).

Two striking differences between broad cortical ExNs and InNs were apparent. First, ExNs had many more positive aDAGs with age (Fig. 4b). Second, we did not observe any significant ExN broad type enrichments (Supplementary Information). Thus, we next examined each of the seven subtypes of ExN and found significant enrichments in *LAMP5*^+^ (layers 1–2), *THEMIS*^+^ (layer 5–6) and *RORB*^+^ ExNs (layers 3–6; Fig. 4c; Supplementary Information)^17^. Surprisingly, although *FEZF2*+ ExN had the highest abundance of aDAGs, we did not observe any gene set enrichments, suggesting broad, non-specific effects of aging on ExN. Notably, ExN and InN enrichment categories differed (Fig. 3c, 4c). ExN subtype enrichment pathways included ‘axonogenesis (GO:0007409)’ and ‘neuron projection (GO:0043005)’ as well as cell-cell adhesion pathways (Fig. 4c–e). Specifically, we observe differential association of semaphorins, SLIT guidance ligands and SLIT and NTRK-like family members, cadherins, and ephrins (Fig. 4d–e; Supplemental Table 4). Interestingly, while some enrichment pathways are shared between ExN subtypes, many aDAGs within these pathways differ between ExN subtypes (Fig. 4d). In contrast to the negative aDAGs observed in cortical InN, the directionality of age-related aDAGs in ExN subtypes was mixed, which may be due to less robust and more heterogeneous expression of marker genes than InN. Given the general decrease in protein translation-related genes observed in cortical InNs, we wanted to see if cortical ExNs also displayed any decrease in those same genes, despite the absence of enrichment for this category. With the exception of *RPS5* in *FEZF2*^+^ ExN, we did not observe any ‘protein targeting to ER (GO:0045047)’ genes differentially associated with age in ExN (Fig. 4f).

ExN subtype aDAGs significantly overlapped with “Aging and Disease Perturbations” from GEO gene sets. Of particular relevance: young vs. aged human frontal cortex, AD, HD, and Lewy Body Dementia (Supplemental Fig. 4e, Supplemental Fig 7f; Supplementary Information). Collectively, these results suggest that aging differentially affects ExN and InN in the number and strength of aDAGs and in the functional categories to which those aDAGs belong.

### OPC-specific negative association of myelination transcription factors with aging in absence of changes in oligodendrocyte lineage cell proportions

**Figure 5:**
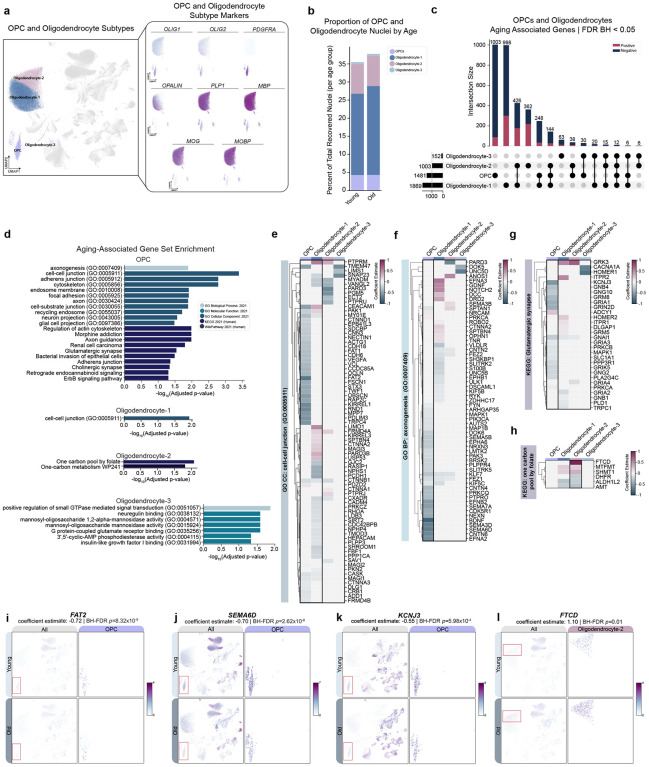
Aging-associated changes in gene expression in oligodendrocyte precursors and oligodendrocytes suggest dysregulation of axon guidance **(a)** UMAP of cell type annotations (left) and feature plots depicting marker genes used to classify OPCs and oligodendrocyte subtypes (right). **(b)** Cell type proportions of OPCs and oligodendrocytes between age groups (see Supplemental Table 5). Oligodendrocyte lineage cells comprise ~35% of total recovered nuclei from both young and old individuals. **(c)** UpSet plot depicting unique vs. shared aDAGs across OPCs and oligodendrocyte subtypes. Cell type sets ordered by cardinality. **(d)** Aging-associated GSE in OPCs and oligodendrocyte subtypes, colored by GO term sets. **(e)** Clustermap of significant aDAGs in OPCs and Oligodendrocytes under the term “GO CC: cell-cell junction (GO:0005911).” **(f)** Clustermap comparing significant aDAGs in OPCs and Oligodendrocytes under the category of “GO BP: axonogenesis (GO:0007409).” **(g)** Clustermap comparing significant aDAGs in OPCs and Oligodendrocytes under the category of “KEGG: Glutamatergic synapse.” **(h)** Clustermap comparing significant aDAGs in OPCs and Oligodendrocytes under the category of “KEGG: one carbon pool by folate.” **(i)** Feature scatters demonstrating decreased expression and association of *FAT2*, **(j)** *SEMA6D*, **(k)** *KCNJ3* in aged OPCs. **(l)** Feature scatter demonstrating increased expression and association of *FTCD* in Oligodendrocyte-2. **Abbreviations**: aDAG, aging-differentially associated gene; BP, biological process; CC, cellular component; *FAT2*, FAT atypical cadherin 2; *FTCD*, formimidoyltransferase cyclodeaminase; GO, gene ontology; *KCNJ3*, potassium inwardly rectifying channel subfamily J member 3; KEGG, Kyoto Encyclopedia of Genes and Genomes; MF, molecular function; OPC, oligodendrocyte precursor cell; *SEMA6D*, semaphorin 6D, UMAP, uniform manifold approximation and projection.

Many studies have documented aging-associated decreases in white matter volume and myelin integrity with age^20,21^. Therefore, we investigated cell-type proportions and aDAGs in oligodendrocyte lineage cell types (Fig. 5a). Unexpectedly, we did not observe a significant shift in oligodendrocyte lineage cell proportions with age overall or within brain regions (Fig. 5b, Supplemental Table 5). Therefore, we next examined the top aDAGs and associated enrichments across these cell types. Similar to previous observations, >50% of top aDAGs in either direction are noncoding (Supplemental Fig. 5a). Only five aDAGs shared between mature oligodendrocytes and three aDAGs shared across all four cell types (Fig. 5c).

We next examined the effect of age on CNS myelination genes derived from the GO term “central nervous system myelination (GO:0022010)” and several of the most abundantly expressed myelination genes (*MBP*, *PLP1*, *MOBP* and 2’,3’-cyclic nucleotide 3’ phosphodiesterase *CNP*). In OPCs, we observed a strong negative association of Hes family bHLH transcription factor 5 (*HES5*), a transcription factor regulating myelination, and weaker negative associations of SRY-box transcription factor 10 (*SOX10*) and teneurin transmembrane protein 4 (*TENM4*, Supplemental Fig. 5b). Surprisingly, we observed very weak negative associations of *MBP* in oligodendrocytes as a broad type (coefficient est.= −0.04, FDR-BH *p=*9.26×10^−4^, SE= 0.009) and in cluster-specific Oligodendrocyte-1 coefficient est.= −0.04, FDR-BH *p*=4.03×10^−4^, SE=0.009) and Oligodendrocyte-2 (coefficient est. = −0.02, FDR-BH *p=*7.61×10^−5^, SE=0.005). PLP1 also had a weak association with age in only Oligodendrocyte-2 (coefficient est = −0.06, FDR-BH *p*= 2.02×10^−3^, SE=0.01; Supplemental Fig. 5b). MOBP and CNP did not have a significant association with age in any cell subtype (Supplemental Table 4). We next investigated gene set enrichment for oligodendrocytes as a broad type followed by each oligodendrocyte subtype. Mature oligodendrocyte aDAGs were only enriched for the “GO cellular component: cell-cell junction (GO:0005911)” category (Supplementary Information). However, when we analyzed oligodendrocyte subtype-specific aDAGs, we observed enrichments for all subtypes (Supplementary Information; Fig. 5d). We examined aDAGs falling under the GO cellular component category “cell-cell junction (GO:0005911)” since this gene set was significant for both OPCs and Oligodendrocyte-1 (Fig. 5d). Among significant aDAGs in this category are cadherins and catenins (Fig. 5e,i). Interestingly, most aDAGs in this shared enrichment category differ between the two cell types, with more OPC aDAGs showing a negative association with age (Fig. 5e).

OPC aDAGs were also enriched for genes in GO: biological process “axonogenesis (GO:0007499)” and “KEGG: Glutamatergic synapse,” the majority of which had negative associations with age (Fig. 5f–g). We observed a negative association of axonogenesis gene families, including semaphorins, contactins, and ephrins (Fig. 5f–j). Notably, these gene families were also differentially associated with age in subsets of ExNs (Fig. 4d). Under “KEGG: Glutamatergic synapse”, we observed negative associations with guanine nucleotide-binding protein subunit genes, G protein-activated inward rectifier potassium channel 1, and glutamate receptors (Fig. 5g,k). Oligodendrocyte-2 enrichments consisted of one-carbon metabolism genes with mixed directionality (Fig. 5h,l). Additionally, we observed aDAGs in OPCs and oligodendrocyte subtypes populated numerous enrichments associated with Aging and Disease Perturbations from GEO gene sets. The majority of these enrichments overlapped with human cancer gene sets, such as astrocytoma, glioblastoma, and oligodendroglioma. We additionally observed overlap with gene sets from aging mouse and human brain, and human-specific AD, MS, PD, and bipolar disorder (Supplemental Fig. 5c).

### Inflammation-associated genes and disrupted fractalkine genes in microglia and astrocytes

**Figure 6:**
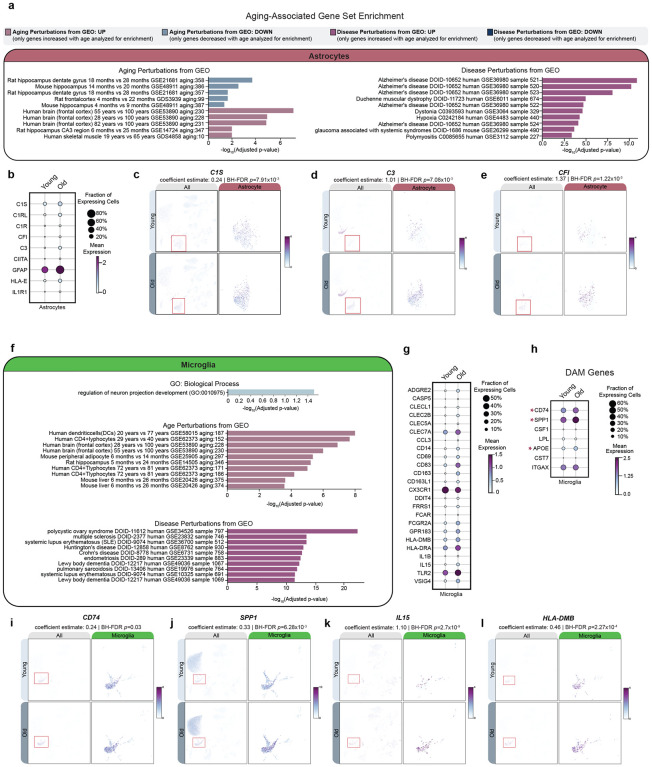
Differentially associated genes with age in astrocytes and microglia overlap with immune response, aging and disease gene sets **(a)** GSE of aDAGs in astrocytes for Aging and Disease Perturbations from GEO gene sets. Top 10 most significant GO terms are shown. For a full list, see Supplementary Information. **(b)** Dotplot of increased expression and/or percentage of astrocytes expressing complement, immune, and gliosis genes. **(c)** Feature scatter showing increased expression and association of complement pathway genes *C1S*, **(d)** *C3*, and **(e)** *CFI* in aged astrocytes. **(f)** GSE of aDAGs in microglia for “GO: Biological Process” and “Aging and Disease Perturbations from GEO” gene sets. Top 10 most significant GO terms are shown. For a full list, see Supplementary Information. **(g)** Dotplot showing significant changes in expression and/or percentage of microglia expressing immune response or **(h)** DAM genes **(i)** Feature plots showing significant increases and associations of *CD74*, **(j)** *SPP1*, **(k)** *IL15*, and **(l)** *HLA-DMB* in aged microglia. **Abbreviations**: aDAG, aging-differentially associated gene; BP, biological process; *C1S*, complement component 1, S subcomponent; *C3*, complement C3; *CD74*, CD74 Antigen (invariant polypeptide of major histocompatibility complex, class II antigen-associated); *CFI*, complement factor I; GEO, Gene Expression Omnibus; GSE, gene set enrichment; *HLA-DMB*, major histocompatibility complex, class II, DM beta; *IL15*, interleukin 15; *SPP1*, secreted phosphoprotein 1

Converging evidence from human genetics and animal models has demonstrated that inflammation can act as a risk factor for aging-related neurodegenerative diseases^22–25^. Therefore, we next investigated top aging aDAGs and GO enrichments in microglia and astrocytes. Of the 797 total microglial aDAGs and 902 astrocyte aDAGs (Supplemental Table 2), <10% are similarly differentially associated with age between the two cell types (Supplemental Fig. 6a–b). However, we noted aDAGs in both cell types share similar ontogeny enrichments for aging gene sets from human frontal cortex and disease gene sets from related human neurodegenerative disorders, including AD, dystonia, multiple sclerosis (MS), Lewy body dementia (LBD) and HD (Fig. 6a,f).

Several complement pathway genes were positively associated with age in astrocytes; specifically *C1S*, *C1RL*, *C1R*, *CFI*, and *C3*^26^ (Fig. 6a–e). C3 is particularly interesting given that it is most abundantly expressed in microglia but only has a differential association with age in astrocytes. Other notable reactive astrocyte genes with significant, age-related positive associations include *CIITA*, *GFAP*, *HLA-E*, and *IL1R1*^27^ (Fig 6b). While we failed to observe enrichments for biological process categories related to immune response in microglia, we did note significant overlap with gene sets from aged human frontal cortex, dendritic cells, and CD4+ T lymphocytes (Fig. 6f). Immune response genes previously documented as increased in aging and/or neurodegeneration were also positively associated with age in microglia in this dataset, notably *IL-15*^28,29^, *HLA-DMB*^26^, *IL-1β*^22,26,30,31^, and *TLR2*^26,32–34^ (Fig. 6g,k–l). We also observed positive associations for several disease-associated microglia (DAM) genes^35^, specifically *CD74*, *SPP1*, and *APOE* (Fig. 6h–j). Finally, we observed a negative association of fractalkine receptor gene *CX3CR1* in microglia and its ligand (*CX3CL1*) in excitatory, inhibitory, and SPN-D1 neurons (Fig. 6g, Supplemental Table 4). This ligand-receptor interaction has been studied extensively in animal models of neurodegeneration and suggested to suppress the production of proinflammatory cytokines^36^. Our results also suggest that neuron-glia signaling may be disrupted with age, which may contribute to immune dysregulation.

### Senescence

Accumulation of senescent cells has been suggested to contribute to age-related neurological decline, which is particularly problematic for the CNS given the long-lived, post-mitotic state of neurons and limited regenerative capacity of the adult brain. We next asked whether there is an increase in the proportions of senescent cells in healthy aged donors. As there is no consensus on a CNS-specific senescence signature, we assessed how multiple senescence-associated gene sets^35,37–39^ change with age across brain regions and broad cell types. We also included the DAM gene set, given it is tuned to a specific brain cell type and that we have demonstrated that several of these genes have positive associations with age in microglia (Fig. 6h).

**Figure 7:**
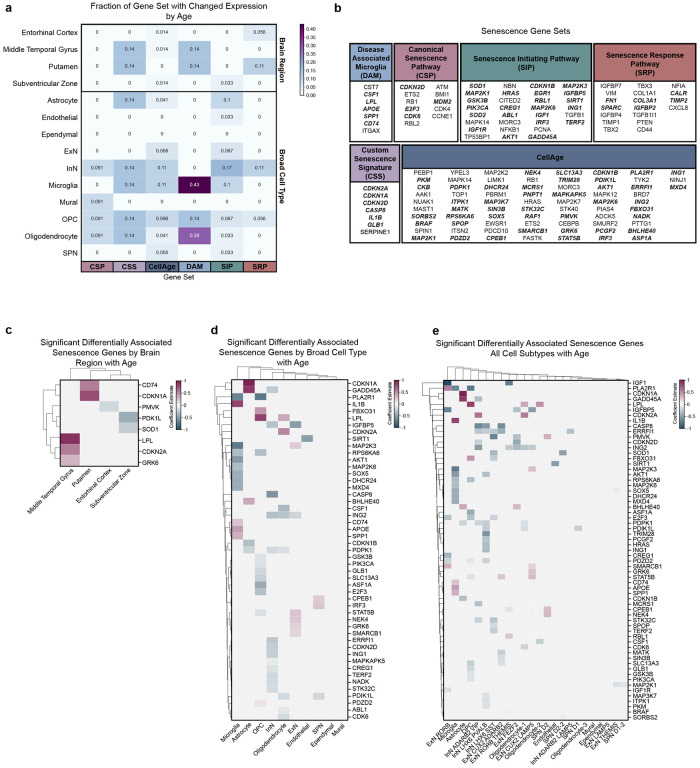
Scoring of senescence-associated gene sets at the level of brain region and broad cell types **(a)** Fraction of senescence associated gene sets changed with age across brain region and broad cell types **(b)** Genes comprising senescence-associated gene sets used for scoring in (a). Genes in bold italics denote significant aDAGs **(c)** Clustermap of significant aDAGs at the level of brain region with strength of association enumerated as coefficient estimate (magenta, significant positive association with age; blue, significant negative association with age). Brain regions ordered by hierarchical clustering. **(d)** Clustermap of significant senescence-related aDAGs at the level of broad type. Broad types ordered by hierarchical clustering. **(e)** Clustermap of significant senescence-related aDAGs at the level of cell subtypes. Cell types ordered by hierarchical clustering. **Abbreviations**: CSP, canonical senescence pathway; CSS, custom senescence signature; DAM, disease-associated microglia; SIP, senescence initiating pathway; SRP, senescence response pathway

At the level of brain region, 11–14% of 3 senescence gene sets exhibit significantly changed expression with age in the PUT, followed by the MTG (1–14% across 3/6 gene sets; Fig 7a–c). Of the broad cell types examined, OPCs exhibited expression changes in 5–15% of genes across all 6 senescence gene sets, followed by oligodendrocytes (3–29% across 5/6 gene sets), inhibitory neurons (9–17% across 5/6 gene sets), and microglia (10–43% across 4/6 gene sets; Fig. 7a–b,d). Senescence-associated genes, which were also significantly differentially associated with age, were then examined, with the number of significant aging aDAGs increasing in parallel with level of cell type resolution (Fig 7c–e).

Additionally, we examined aDAGs from cell types which exhibited significant enrichment from previous human aging datasets. Interestingly, microglia and astrocytes were significantly enriched for positive aDAGs previously shown to be increased in the aged frontal cortex, including *SERPINA3*, *IFITM2*, *CD163*, and *CD14* (Supplemental Fig. 7a–e). In contrast, cell types enriched for genes decreased in aged human frontal cortex, including *KCNF1*, *LY6H*, and *PFN1*, were limited to neurons (Supplemental Fig. 7f–i).

### Cell type-specific differential associations of genes nearest to GWAS-identified risk loci: Alzheimer’s disease and Parkinson’s disease

Given that aging is a risk factor for many NDDs, we next examined genes nearest to genome-wide association study (GWAS) risk loci for Alzheimer’s disease (AD) and Parkinson’s disease (PD). We first examined the baseline mean expression of AD- and PD-associated genes across brain regions and cell types (Supplemental Fig. 8a–d). While many genes are broadly expressed across multiple cell types, several exhibit cell type-specificity^40^. Therefore we next examined if these genes exhibited significant associations with age in a brain region-and/or cell type-specific manner.

**Figure 8:**
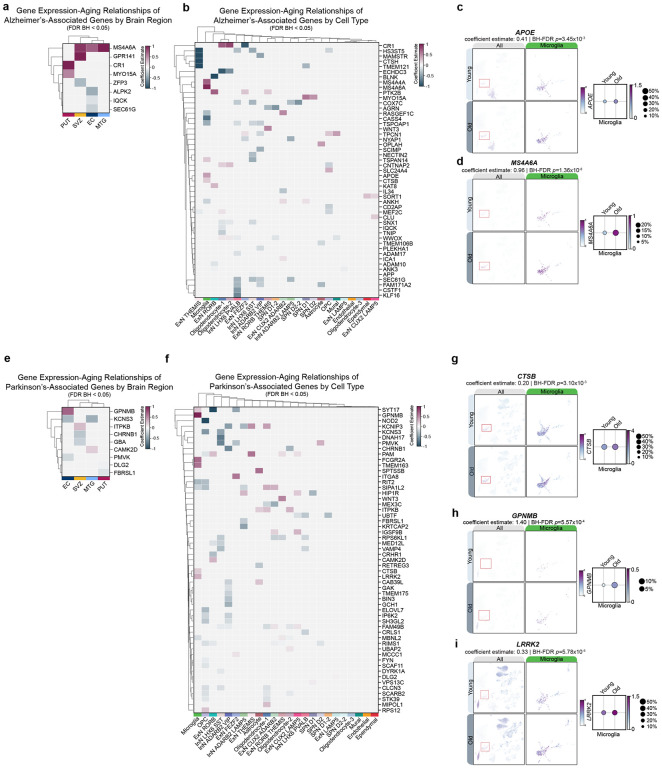
Aging-associated changes in genes nearest to GWAS-identified risk loci for Alzheimer’s disease and Parkinson’s disease **(a)** Clustermap of significant aDAGs which overlap with genes nearest to AD GWAS-nominated risk loci at the level of brain region. Strength of association enumerated as coefficient estimate (magenta, significant positive association with age; blue, significant negative association with age). Brain regions ordered by hierarchical clustering. **(b)** Clustermap of significant aDAGs which overlap with genes nearest to AD GWAS-nominated risk loci across cell types. Cell types ordered by hierarchical clustering. **(c)** Feature scatter and dot plot showing increased expression and association of *APOE* and **(d)** *MS4A6A* with age in microglia **(e)** Clustermap of significant aDAGs which overlap with genes nearest to PD GWAS-nominated risk loci across brain regions. Brain regions ordered by hierarchical clustering. **(f)** Clustermap of significant aDAGs which overlap with genes nearest to PD GWAS-nominated risk loci across cell types. Cell types ordered by hierarchical clustering. **(g)** Feature scatter and dot plot showing increased expression and association of *CTSB*, **(h)** *GPNMB*, and **(i)** *LRRK2* with age in microglia **Abbreviations**: aDAG, aging-differentially associated gene; AD, Alzheimer’s disease; *APOE*, apolipoprotein E; *CTSB*, cathepsin B; *GPNMB*, glycoprotein nmb; GWAS, genome-wide association study; *LRRK2*, leucine-rich repeat kinase 2; *MS4A6A*, membrane spanning 4-domains A6A; PD, Parkinson’s disease

We detected expression of 110/112 AD GWAS-nominated genes^41^ in this dataset (Supplemental Fig. 8a–b), of which 52 were significantly differentially associated with age (Fig. 8a–b). Of particular interest is apolipoprotein E (*APOE*), variants of which can increase risk for developing late-onset AD (LOAD) and Lewy body dementia. We noted a microglia-specific increase in the percentage of cells expressing *APOE* and concomitant increase in level of expression, as well as a significant positive association with age (coefficient est.=0.41, BH-FDR p=3.45×10^−3^, SE=0.10; Fig. 8a–c). Interestingly, we did not observe a significant age association of *APOE* in astrocytes, where it is abundantly expressed (Fig. 8b–c; Supplemental Fig. 8b). We additionally observed a strong positive association of membrane spanning 4-domains A6A (*MS4A6A*) with age in all brain regions except the putamen (Fig. 8a). We note that the positive association of *MS4A6A* at the brain region level is driven by an increase in the number of microglia expressing *MS4A6A*, the strength of its expression in microglia, and a strong positive association with age exclusively in microglia (coefficient est.= 0.96, FDR-BH *p*=1.36×10^−8^, SE=0.13; Figure 8a–b, d).

Other notable aDAGs include negative age associations of endoplasmic reticulum protein translocation complex gene *SEC61G* and mitochondrial electron transport chain enzyme component cytochrome c oxidase subunit 7C (*COX7C)* in multiple cortical inhibitory neuron subtypes, fatty acid biosynthesis gene *ECHDC3* in oligodendrocytes, and a positive association of complement C3b/C4b receptor 1 (*CR1*) in oligodendrocyte-1 and oligodendrocyte-2 (Fig. 8b).

Next, we examined aging associations of 82/87 detected genes nearest to PD GWAS-identified risk loci^42^ and observed that 56/82 genes had a significant association with age, most strikingly in OPCs and microglia (Supplemental Fig. 8c–d; Fig. 8e–f). Notable genes which exhibited microglia-specific differential associations include cathepsin B (*CTSB*; coefficient est.= 0.20, FDR-BH *p*=3.14×10^−3^, SE=0.05) glycoprotein Nmb (*GPNMB*; coefficient est.= 1.40, FDR-BH *p*=5.57×10^−4^, SE=0.30), and leucine-rich repeat kinase 2 (*LRRK2*; coefficient estimate: 0.33, FDR-BH *p*=5.78×10^−5^, SE=0.06; Fig. 8f–i). Of microglia-specific aDAGs, *GPNMB* had a strong positive association with age, despite being more highly expressed in OPCs and mural cells (Fig. 8e,h). Similarly, *LRRK2* exhibited a significant positive association in microglia, despite being most highly expressed in OPCs (Fig. 8e,i). Other notable aDAGs shared across multiple cell types include signal induced proliferation associated 1 like 2 (*SIPA1L2)*, potassium voltage-gated channel interacting protein 3 (*KCNIP3*), and peptidyl-glycine alpha-amidating monooxygenase (*PAM*; Fig. 8f).

These results collectively suggest that a large subset of genes nominated by AD (52/112 genes) and PD (56/87 genes) GWAS are differentially expressed with aging, independently of disease. Many of these differential associations occur in microglia and OPCs. Additionally, we demonstrate the utility of snRNAseq in detecting moderate, cell type-specific associations with age that otherwise would be masked using bulk strategies.

## Discussion

Here, we performed snRNAseq on nuclei from 12 individuals without a clinical or pathological history of neurodegenerative disease to examine changes in cell type populations and transcriptomes during healthy aging across multiple brain regions. We isolated nuclei from the entorhinal cortex, middle temporal gyrus, putamen, and subventricular zone, which are each differentially affected in neurodegenerative diseases. In total, we annotated 155,192 nuclei comprising all expected neuronal and non-neuronal cell types and subtypes (Fig. 1).

While we failed to observe any significant changes in cell type proportions (Fig. 2), we noted striking diverging transcriptome patterns across brain regions and cell types (Supplemental Fig. 2). We observed that >50% of aging differentially associated genes (aDAGs) in each brain region, broad cell type, and cell subtype are unique with very few aDAGs shared across brain regions or cell types. At the level of brain regions, we observed predominantly more positive aDAGs in the MTG and PUT, and more negative aDAGs in the SVZ and EC, with only one aDAG shared across all regions. At the level of broad cell types, we observed predominantly negative aDAGs in InNs and OPCs, with all other broad type aDAGs exhibiting mixed directionality.

While these results suggest the absence of a global, shared aging gene signature, we noted that over 50% of the top aDAGS were annotated as lncRNAs or pseudogenes. Two of the most commonly differentially associated lnRNAs across cell types in the present dataset are *FAM66E* and *LERFS*, whose downregulation has been documented in human lung adenocarcinoma^43^ and rheumatoid arthritis^44^, respectively. However, their function in the brain is unknown. Studies have suggested a regulatory role for lncRNAs in the contexts of alternative splicing, telomere stability, cell proliferation, intercellular communication, proteostasis, and epigenetic regulation; processes which are known to be altered during the course of aging and in neurodegeneration^45–48^. Given the abundance and diversity of lncRNAs in the CNS, further investigation into their role in aging and disease is warranted.

We next investigated aDAGs in InN and ExN subtypes and similarly observed that >50% of aDAGs for a given cell subtype were unique. We found striking differences between cortical and subcortical InNs, with cortical InNs exhibiting decreased associations of genes involved in protein synthesis and targeting, gene expression, and lncRNA processing, with *LHX6*^+^*PVALB*^+^ being most strongly affected (Fig. 3). aDAGs in ExNs, in contrast, were far fewer in number with more mixed directionality and categorically different GO enrichments, including axonogenesis, cell adhesion, and cell communication by electrical coupling (Fig. 4). The difference in strength and number of aDAGs in InNs vs. ExNs is consistent with previous studies demonstrating selective vulnerability of *LHX6*^+^*PVALB*^+^ and *LHX6*^+^*SST*^+^ cortical neurons in the EC and MTG, respectively, in AD. Given that disrupted proteostasis is a major feature of AD, our data suggest decreases of protein synthesis and targeting genes in these cell types *en masse* during normal, healthy aging may set the stage for selective vulnerability of these InNs and subsequent imbalance of inhibitory-excitatory signaling observed in AD^49,50^.

Changes in white matter integrity^20,21^ and expression of myelination genes^51^ have been documented in previous studies of aging and neurodegeneration in animal models and humans. Surprisingly, we did not observe a change in OPC or oligodendrocyte numbers between young and old individuals in any brain region (Supplemental Table 5). However, we demonstrate OPC-specific negative associations of development and myelin gene-associated transcription factors *HES5*, *SOX10*, and *NKX2–2*^52–54^ as well as teneurin transmembrane protein 4 (*TENM4*), with only negligible negative associations of *MBP* and *PLP1* in oligodendrocyte-1 and oligodendrocyte-2, respectively (Supplemental Fig. 5; Supplemental Table 4). While our data suggest that terminal differentiation of OPCs to oligodendrocytes, upstream transcription factors regulating myelination, and support of axonogenesis may be disrupted with age in absence of cell loss, we are unable to rule out post-transcriptional and post-translational contributions to white matter integrity disruption.

Previous studies examining brain aging using bulk RNAseq have consistently demonstrated altered inflammatory signaling with age. While we failed to observe GO enrichments related to inflammation in any brain region, broad type, or cell subtype, we did observe that the majority of the top protein-coding aDAGs across brain regions were associated with inflammation (Supplemental Fig. 2c). In astrocytes, we observed positive associations of reactive gliosis genes and multiple complement pathway genes, the latter of which is particularly interesting given the complement system’s role in synaptic pruning during development and its potential involvement in elimination of aberrant synapses in neurodegenerative diseases^43–48^. In microglia, we observed positive associations of proinflammatory cytokine, major histocompatibility complex, and disease-associated microglia genes. Moreover, we noted a decreased association of fractalkine ligand and receptor (CX3CL1-CX3CR1) genes in neurons and microglia, respectively (Fig. 6; Supplemental Table 4). Our results suggest that inflammatory genes are increased with age and that homeostatic neuron-glia signaling may be disrupted, thus contributing to chronic immune dysregulation and synapse elimination seen in both aging and neurodegenerative diseases.

Importantly, we found significant overlap with previous mouse, non-human primate, and human brain aging and neurodegeneration datasets. Examples include a strong positive association of protease *RHBDL3* in ExN subtypes, previously shown to be strongly increased with age in the human frontal cortex^6,19^, and negative associations of *NPY* and its receptor *NPYR1* in the MTG, SVZ, subsets of ExN, and OPCs. We additionally replicated an aging-associated decrease in neuropeptide *SST*^6,19^ in both the EC and MTG, and in *ADARB2*^+^*LAMP5*^+^ cortical InNs, noting negative associations of related receptors *SSTR1* and *SSTR2* across cortical InNs, ExNs, and mural cells. When comparing aDAGs from this study to human aging datasets, we note that cell types with significant enrichments for genes increased with age included astrocytes and microglia, while cell types with significant enrichments for genes decreased with age were restricted to neuron subtypes (Supplemental Fig. 7).

Notably, these differential associations occur in absence of any widespread overt loss or gain of cell types with age (Fig. 2, Supplemental Fig. 2). These findings demonstrate the utility and robustness of snRNAseq in 1) replicating previous findings from bulk RNAseq; 2) providing increased resolution to disambiguate changes in gene expression from cell loss; and 3) determining which particular cell types significant transcriptional changes are occurring.

Our analysis of senescence gene sets is consistent with previous senescence studies in postmortem brain tissue. The percentage of senescent cells is dependent on which gene set is used. While OPCs appear to have differential expression of senescence markers across all gene sets, consistent with results from mouse models of amyloid-beta accumulation and post-mortem human AD brain^61^, further investigation is needed to define cell type-specific senescence signatures in the CNS, particularly in post-mitotic neurons (Fig. 7).

Finally, we examined the relationship between aging and the expression of genes nearest to GWAS-identified risk loci for AD and PD. Many of these disease-associated genes had a significant age-association specific to a single cell type, despite being more abundantly expressed in other cell types at baseline. For example, AD-associated *APOE* and PD-associated *LRRK2*^62,63^ and *GPNMB*^64,65^ are positively associated with age in microglia, despite being more highly expressed in astrocytes and OPCs, respectively (Fig. 8). Our data provide context for and highlight the necessity of testing GWAS-nominated disease risk genes in relevant subtypes for preclinical mechanistic studies *in vitro* and *in vivo*, and subsequently, for developing more cell type-specific, targeted therapeutic strategies.

We note several limitations of our study. First, we examined aging as a categorical rather than continuous variable, in part due to the multiregion/donor design of the study. Given that the prodromal period for age-related neurodegenerative diseases is estimated to be >10 years, examining individuals aged 40–60 could be particularly insightful. While we cannot rule out the possibility that donors would eventually go on to develop dementia or other NDDs given a long prodrome, tissue was selected based on lack of clinical and postmortem neuropathological criteria consistent with NDDs. Second, while we balanced each age group by sex, we were not sufficiently powered to examine a gene expression*sex*age interaction. This analysis could be particularly informative in the context of neurodegenerative diseases like PD and AD, which differentially affect males and females, respectively.

In summary, the current dataset provides a novel resolution of the effects of chronological aging across brain regions and cell types. Surprisingly, using unbiased approaches, we show that multiple aDAGS are also genes that modulate risk of NDDs. These data support that future studies should be directed to larger sample series and incorporate additional single cell measures of the effects of aging in the healthy brain.

## Methods

### Isolation of nuclei from human brain

Nuclei were isolated using the Nuclei PURE Prep Nuclei Isolation Kit (Sigma #NUC201) per the manufacturer’s instructions with slight modifications. To streamline workflow and decrease potential batch effects, we used a pooling approach prior to library preparation followed by post-sequencing demultiplexing, resulting in 6 pools of 8 samples each. Each pool was balanced for age group and sex. Of note, no single pool contained tissue from 2 regions from the same donor.

A 100mg piece of tissue was placed on a fresh, pre-chilled petri dish, trimmed, and weighed. Approximately 25 mg tissue/sample for a total of 8 samples per pool was combined and homogenized in a single cold douncer containing 2 ml ice-cold lysis buffer (Nuclei PURE Lysis Buffer (Sigma #L9286), 0.1M DTT from freshly thawed aliquot (Sigma #GE17-1318-01), and 0.1% Triton X-100 (Thermofisher Scientific #T1565) Tissue was homogenized with 25 strokes of a loose pestle, and 25 strokes of a tight pestle before being transferred to a tube containing 8ml cold lysis buffer, vortexed 2–3s, and left on ice for 10 minutes. Following lysis, cold 1.8M sucrose cushion solution (Nuclei PURE 2M Sucrose Cushion Solution (Sigma #S9308), Nuclei PURE Sucrose Cushion Buffer (Sigma #S9058) and 0.1M DTT) was added to the bottom of an ultracentrifuge tube (Beckman Coulter #344058) on ice. To each lysate, cold 1.8M sucrose cushion solution was added and mixed using a serological pipette. The lysate solution was slowly layered on top of the sucrose cushion, placed in a precooled ultracentrifuge, and centrifuged for 45 min. at 30,000 × *g* at 4°C.

Sample tubes were removed from the ultracentrifuge and placed on ice. The supernatant was aspirated completely, and the nuclei pellet was resuspended in Nuclei Suspension Buffer on ice (NSB; 1ml cold PBS (Thermofisher Scientific #10010–023), 0.01% BSA (New England Biolabs #B9000S), 0.1% SUPERase RNase inhibitor (Thermofisher Scientific #AM2696), transferred to a 15ml tube containing an additional 4ml NSB buffer, mixed, and washed by centrifugation at 500 × *g* for 5 min. at 4°C. The pellet was resuspended in 1 ml NSB, filtered through a 70μM Cell Strainer (STEMCELL Technologies #27216) to remove debris, and washed again by centrifugation. The supernatant was aspirated, and nuclei were resuspended in a final volume of 110μl.

To determine nuclei concentration, Acridine Orange/Propidium Iodide (Logos Biosystems #F23001) was added to nuclei suspension in a separate tube and counted using a LUNA-FL Dual Fluorescence Cell Counter (Logos Biosystems). An appropriate volume of nuclei was diluted with NSB to achieve a final single suspension of 80,000 nuclei to achieve maximum nuclei recovery while minimizing the multiplet rate. Approximately 10,000 nuclei from this single suspension were loaded into each of 8 lanes of a 10x Genomics NextGEM Chip G (10x Genomics #1000127) and inserted into a 10xGenomics Chromium Controller (10xGenomics #1000204) according to the manufacturer’s instructions. We loaded 10k nuclei per lane, targeting ~6k nuclei recovered per lane with a multiplet rate of ≤5%. The remainder of library preparation was conducted according to the Chromium Single Cell 3’ Reagent Kits User Guide (v3.1 Chemistry; Rev.D). Final libraries were sequenced at the NIH Intramural Sequencing Center (NISC) at a read depth of 25,000 paired-end reads per nucleus on an Illumina NovaSeq 6000. Data was processed with CellRanger count v5.0.1 with refdata-gex-GRCh38–2020-A as the reference, with introns included.

### Genotype preparation

Genotypes were assayed from three array-based genotyping platforms: Human1M-Duov3_B, HumanHap650Yv3.0, and HumanOmni5-Quad. Genotypes were merged and filtered to variants common to all three platforms and called in 95% of the samples, and that are single-nucleotide variants (SNVs). Genotype files were lifted over from hg19 to hg38. The Plink2^66^, bcftools^67^, and Picard (https://broadinstitute.github.io/picard/) tool sets were used to filter, format, and liftover the genotype files.

### Demultiplexing of Pooled Samples

Pooled samples were demultiplexed using the demuxlet tool^68^. The prepared subject genotypes and the aligned single-nuclei bam files were used to deconvolute the cells’ sample identities. Data were processed on the Google Cloud Platform (GCP) using the Cumulus/Demuxlet workflow (WDL, https://cumulus-doc.readthedocs.io/en/0.12.0/demuxlet.html), which executes the demuxlet tool contained in the Statgen Popcle suite (https://github.com/statgen/popscle). Job submission to GCP for execution was done via the Broad WDL runner (https://github.com/broadinstitute/wdl-runner) and GCP Life Sciences interface (https://cloud.google.com/life-sciences/docs). SCANPY^69^ was used to read in the 10X filtered matrix files into an AnnData object and integrate sample identity for the deconvoluted cells, along with sample information into the ‘obs’ information and then combine all data into a single AnnData object.

### Clustering and cell-type identification

The single-cell analysis tool Pegaus^70^ (https://pegasus.readthedocs.io/en/stable/index.html) was used to combine, filter, normalize, cluster, and perform the initial cell-type identities of the demultiplexed single-nuclei count data. Basic filtering was done with Pegasus excluding cells that did not include at least 200 genes, genes that were not present in at least three cells, and cells that had more than 10% mitochondrial content^71^. Counts were transformed to a total-count normalization of 10,000 reads per cell and log transformation. Highly variable features were used for clustering, 2000 genes. Principal components analysis was used to reduce the dimensionality of the data from 2000 high variable genes to 50 principal components (PC). Batch effects were corrected with Harmony^72^ applied to the 50 PCs. The k-nearest neighbors were computed from the Harmony corrected components based on 100 nearest neighbors for each cell the L2 distance and the hnswlib search algorithm^73^. The Leiden^74^ algorithm was used to identify clusters on the neighborhood graph; multiple resolutions were used after inspection. Differential expression for each cluster against all others along with known marker genes for broad central nervous system cell-types was used to make the initial inferences for each cluster’s putative cell-type. For the differential expression analysis performed with Pegasus the default Mann-Whitney U test was used. Uniform Manifold Approximation and Projection (UMAP)^75^ was used to visualize, inspect, and evaluate the clustering and initial cell-type assignments. Inspection and evaluation of the clusters and their cell-type assignments were performed using dendrograms, dot plots, violin plots, volcano plots, and UMAP scatter plots over combinations of gene makers and sample information entities. After evaluation, a final Leiden resolution of 0.85 was used. During inspection more refined cell-type markers were included and where needed cell-type assignments were corrected. As part of the evaluation and inspection a sub-clustering approach was also used where subsets of cells based on specific clusters were subset and re-clustered. Cell trajectory and diffusion was evaluated using the Force-directed Layout^76^ algorithm available in the Pegasus tool. Three clusters (12,753 nuclei), where a determination of the cell-type assignment could not be made via algorithm or manual inspection and evaluation were excluded for further analysis.

### Age-related differential expression and association

Three sets of differential expression analysis were performed with age group as the independent variable. The three sets were brain region, cell-type, cluster specific. Where the difference between cell-type and cluster specific is in instances where multiple clusters had the same cell-type assignment each of these clusters were analyzed separately as well as together as a cell-type. For example, multiple clusters of oligodendrocytes were identified, and each of these were analyzed separately as well as a single cell-type oligodendrocyte. Age was treated as a binary variable representing young or old, where young subjects were 20 to 30 years of age at death while old subjects were 60 to 85 years of age at death. Only genes with non-zero values in at least 3 cells within the individual analysis set and with non-zero cells from at least 50% of the subjects were included in differential expression analysis between the age groups. For computational efficiency, the differential expression analysis was performed in two steps. First, a simple t-test between age groups was performed using the diffxpy package (https://diffxpy.readthedocs.io/en/latest/index.html). Any result where a nominally significant difference was detected was considered for follow-up in a second step. For the second step of the differential expression analysis, in order to address pseudoreplication and zero-inflation that impact analyses in single-cell experiments, we utilized a generalized linear mixed model (GLMM) with a Tweedie distribution^77^. To account for pseudoreplication a fixed-effect term is included to account for the sample, while a Tweedie distribution was specified to account for zero-inflation. Additionally, the pool number was included as a term to account for residual batch effects from pooling that were not corrected by the Harmony batch effects correction step. The glmmTMB^78^ R package was used to run this model; gene ~ age_group + pool + (1|sample_id). To correct for multiple testing within each analysis set the resulting p-values were adjusted using Benjamini and Hochberg false discovery rate (fdr_bh), as implemented in the statsmodel multitest Python package (https://www.statsmodels.org).

### Gene Set Enrichment

To determine biological pathways with significant age associations, we used the GSEApy package^79^ with Enrichr (gseapy.enrichr) to perform overrepresentation analysis on aDAGs determined by glmmTMB (*FDR BH p*< 0.05) for each brain region, broad type, and cell subtype. We queried significant aDAGs against libraries within the ‘Human’ enrichr database: ‘GO_Biological_Process_2021’, ‘GO_Cellular_Component_2021’, ‘GO_Molecular_Function_2021’, ‘KEGG_2021_Human’, ‘WIKIPATHWAYS_2021_Human’. We additionally queried ‘Aging_Perturbations_from_GEO_up, ‘Disease_Perturbations_from_GEO_up,’ ‘Aging_Perturbations_from_GEO_down,’ and ‘Disease_Perturbations_from_GEO_down’ gene sets using positive *or* negative aDAGs only, respectively. A cutoff of FDR < 0.05 was used to display significant terms. All gseapy.enrichr results are available in the supplementary information.

### Senescence Scoring

The score_genes function from SCANPY^69^ was used to score sets of marker genes for both senescence and DAM. The score_genes function generates a score based on the average expression of the provided gene markers against the average expression of a reference set; here all genes were used as the reference set. In these analyses any cell with a score of more than two standard deviations from the mean of a broad cell-type’s scored cells was considered positive. The senescence marker genes included six separate sets and the DAM signature was based on a single set of marker genes. These marker sets are from previously published studies. The DAM signature consisted of six genes^35^. The senescence sets include: custom senescence signature (CSS) comprised of 7 genes^35^, Canonical Senescence Pathway (CSP) using 11 genes, Senescence Response Pathway (SRP) using 18 genes, Senescence Initiating Pathway (SIP) using 30 genes^38^, Cell Age using 73 genes^80^. Cells for each broad cell-type were scored for each senescence marker set and the DAM set was only scored for microglia.

## Supplementary Material

Supplement 1

Supplement 2

Supplement 3

Supplement 4

Supplement 5

Supplement 6

1

## Data Availability

Code: https://github.com/neurogenetics/ADRD_Brain_Aging Summary level results: https://zenodo.org/record/7847472 Individual level data: https://nda.nih.gov/edit_collection.html?id=3151 Streamlit App: https://brainexp-hykyffa56a-uc.a.run.app/

## Highlights

- Establishment of a single nuclei atlas of human aging in four brain regions
- Each region and cell type exhibits a unique aging-associated transcriptome signature
- Gene expression changes occur in absence of overt cell loss and are categorically unique across cell types
- Neurological disease-associated genes have age-associated expression patterns in specific cell types in the context of healthy aging
