# Supplementary material for: Region-Specific Transcriptional Signatures of Brain Aging in the Absence of Neuropathology at the Single-cell Level
Source: bioRxiv. 2025 Aug 19:2023.07.31.551097. Originally published 2023 Aug 1. Preprint. [Version 2] doi: 10.1101/2023.07.31.551097 (PMC10418086; doi:10.1101/2023.07.31.551097)
Supplement: Supplement 10 [file NIHPP2023.07.31.551097v2-supplement-10.pdf]

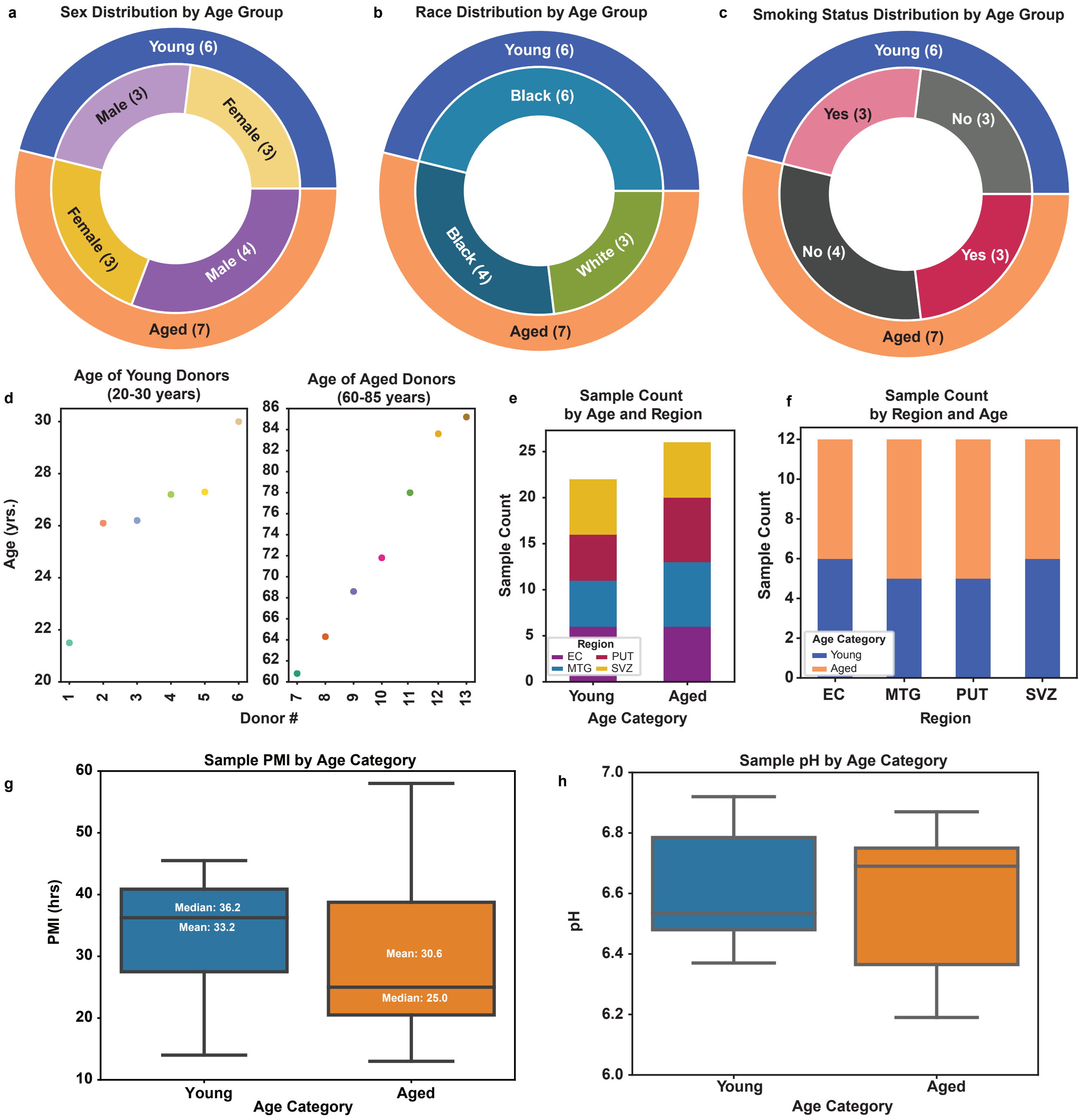
a
Sex Distribution by Age Group
Young (6)
Male (3)
Female (3)
Female (3)
Male (4)
Aged (7)
b
Race Distribution by Age Group
Young (6)
Black (6)
Black (4)
White (3)
Aged (7)
c
Smoking Status Distribution by Age Group
Young (6)
Yes (3)
No (3)
No (4)
Yes (3)
Aged (7)
d
Age of Young Donors (20-30 years)
Age of Aged Donors (60-85 years)
Age (yrs.)
Donor #
e
Sample Count by Age and Region
Sample Count
Region
EC
MTG
PUT
SVZ
Young
Aged
Age Category
f
Sample Count by Region and Age
Sample Count
Age Category
Young
Aged
EC
MTG
PUT
SVZ
Region
g
Sample PMI by Age Category
PMI (hrs)
Median: 36.2
Mean: 33.2
Mean: 30.6
Median: 25.0
Young
Aged
Age Category
h
Sample pH by Age Category
pH
Young
Aged
Age Category

bioRxiv preprint doi: https://doi.org/10.1101/2023.07.31.551097; this version posted August 19, 2025. The copyright holder for this preprint (which was not certified by peer review) is the author/funder. This article is a US Government work. It is not subject to copyright under 17 USC 105 and is also made available for use under a CC0 license.

Supplemental Figure 1: Donor Demographics and Sample Characteristics
Distribution of demographic characteristics across 13 donors within young (20-30 yrs.) versus aged (60-85 yrs.) groups: **(a)** sex, **(b)** race, **(c)** smoking status, and **(d)** age. Distribution of tissue sample counts **(e)** by region within an age category and **(f)** by age category within a region of interest demonstrates a relatively even sample distribution. This sample series was selected based on minimizing variability in **(g)** post-mortem interval (PMI; range 13-58 hrs; mean: 33.2 hrs.; median: 36.2 hrs.) and **(h)** brain pH (range 6.19-6.92).

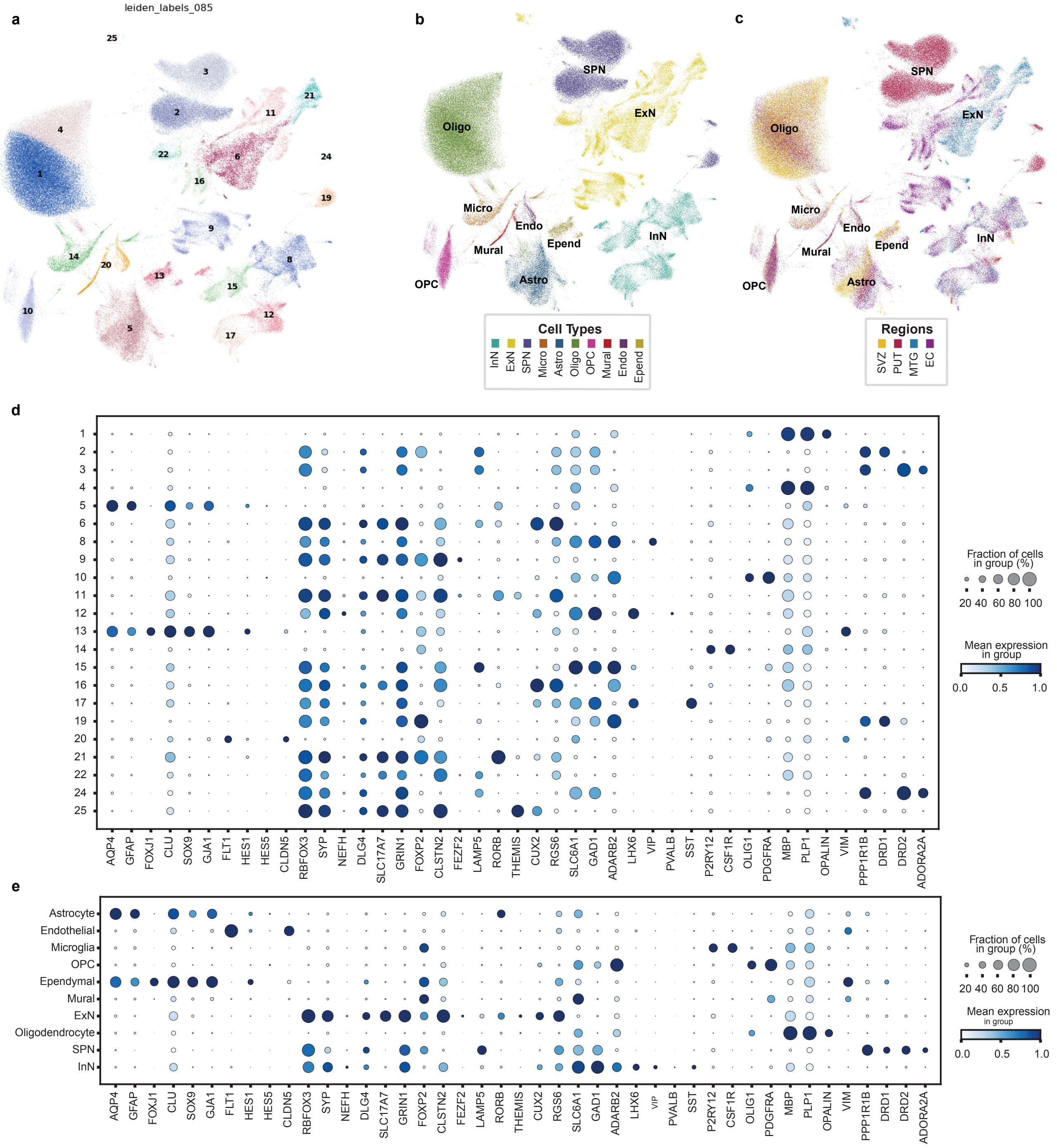

a
leiden_labels_085
b
SPN
ExN
Oligo
Micro
Endo
Epend
Mural
InN
OPC
Astro
Cell Types
InN
ExN
SPN
Micro
Astro
Oligo
OPC
Mural
Endo
Epend
c
Regions
SVZ
PUT
MTG
EC
d
Fraction of cells in group (%)
20 40 60 80 100
Mean expression in group
0.0 0.5 1.0
AQP4 GFAP FOXJ1 CLU SOX9 GJA1 FLT1 HES1 HES5 CLDN5 RBFOX3 SYP NEFH DLG4 SLC17A7 GRIN1 FOXP2 CLSTN2 FEZF2 LAMP5 RORB THEMIS CUX2 RGS6 SLC6A1 GAD1 ADARB2 LHX6 VIP PVALB SST P2RY12 CSF1R OLIG1 PDGFRA MBP PLP1 OPALIN VIM PPP1R1B DRD1 DRD2 ADORA2A
e
Astrocyte
Endothelial
Microglia
OPC
Ependymal
Mural
ExN
Oligodendrocyte
SPN
InN

bioRxiv preprint doi: https://doi.org/10.1101/2023.07.31.551097; this version posted August 19, 2025. The copyright holder for this preprint (which was not certified by peer review) is the author/funder. This article is a US Government work. It is not subject to copyright under 17 USC 105 and is also made available for use under a CC0 license.

Supplemental Figure 2: CNS Marker Gene Expression across 25 Leiden Clusters reveals 10 unique cell-types across four brain regions.
(a) Leiden clustering of 151,647 nuclei at 0.85 resolution resulted in 25 distinct clusters. **(b)** Leiden clusters re-colored and labeled according to broad cell-type annotation and **(c)** brain region of origin. **(d-e)** Relative expression levels and proportion of cell-types expressing canonical marker genes were used to manually annotate to the broad cell-type level.

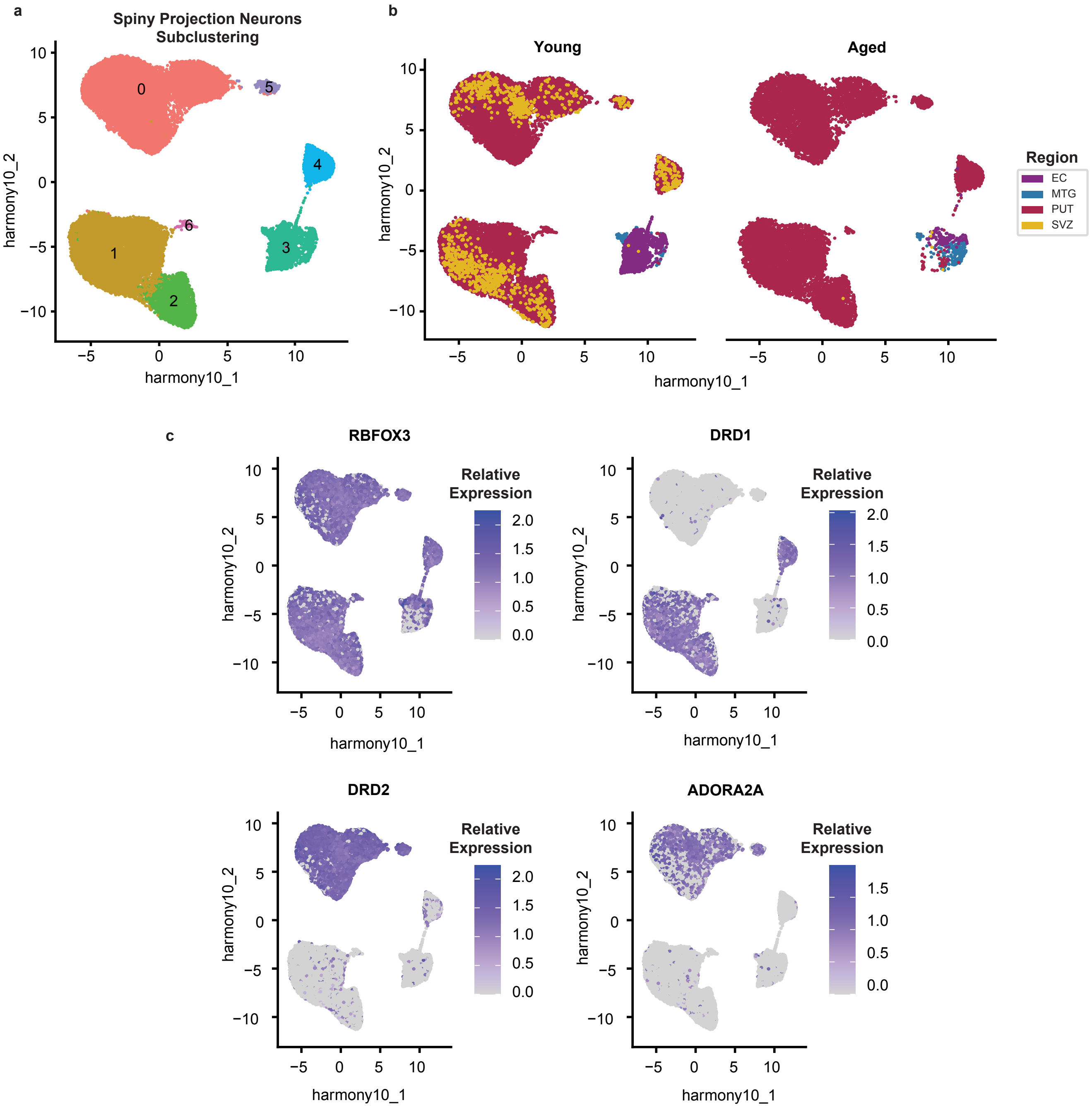

a
Spiny Projection Neurons Subclustering
0
1
2
3
4
5
6
harmony10_1
harmony10_2
b
Young
Aged
Region
EC
MTG
PUT
SVZ
c
RBFOX3
DRD1
DRD2
ADORA2A
Relative Expression

bioRxiv preprint doi: https://doi.org/10.1101/2023.07.31.551097; this version posted August 19, 2025. The copyright holder for this preprint (which was not certified by peer review) is the author/funder. This article is a US Government work. It is not subject to copyright under 17 USC 105 and is also made available for use under a CC0 license.

Supplemental Figure 3: Subclustering of nuclei annotated as spiny projection neurons affirms regional specificity for putamen and indicates probable misannotated nuclei
(a) Subclustering of nuclei annotated as spiny projection neurons in the initial round of clustering results in 7 sub-clusters. **(b)** Separating these nuclei by age group (young, left; aged, right) and coloring them by brain region of origin reveals that subclusters (0-2 and 5-6) largely come from the putamen. Nuclei from other regions were few in number (n=3,545), so they were re-annotated as "Other" and excluded from further analysis. (**c**) Relative normalized expression levels for known SPN marker genes differentiate subclusters and confirm SPN identity.

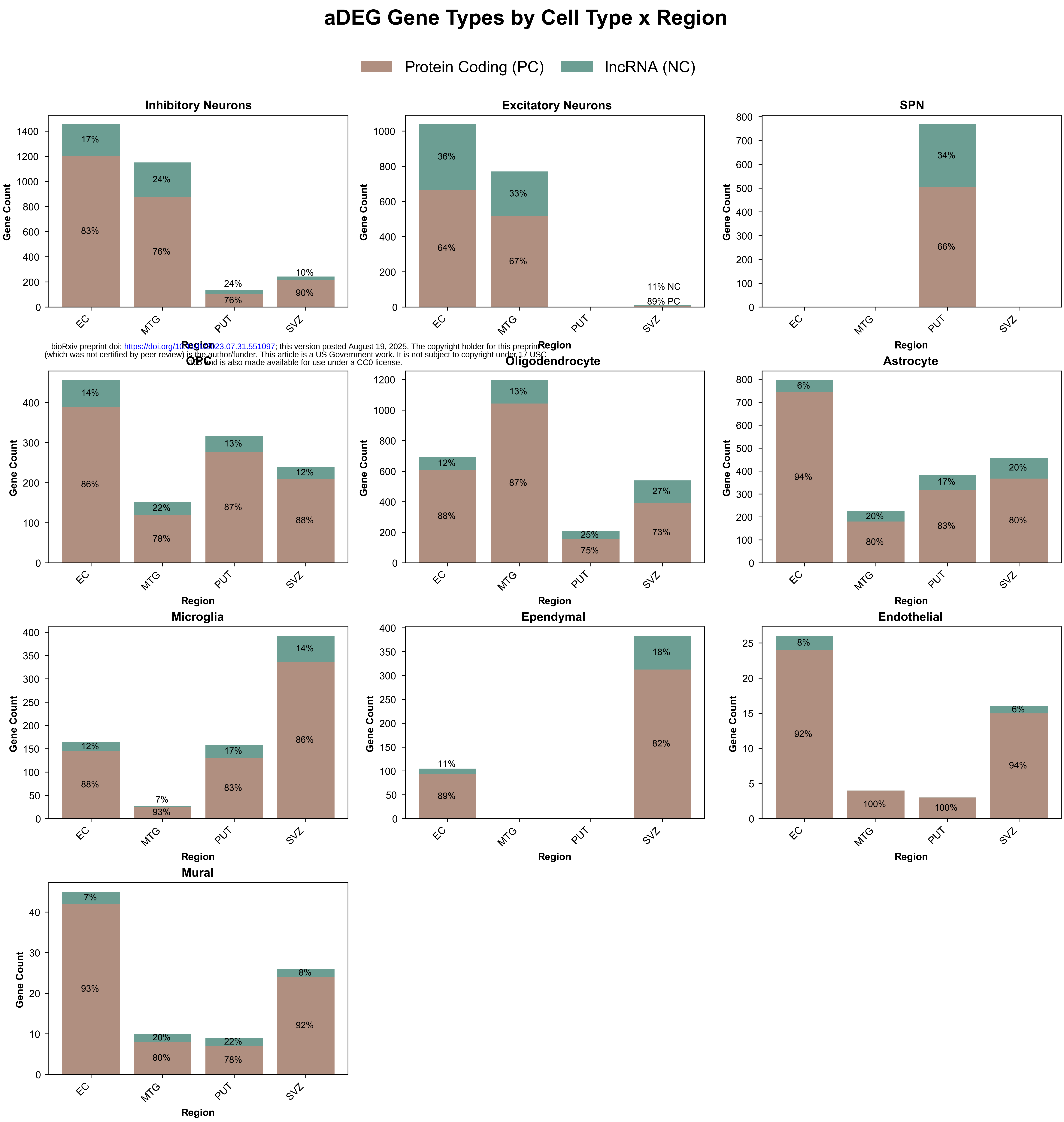

bioRxiv preprint doi: https://doi.org/10.1101/2023.07.31.551097; this version posted August 19, 2025. The copyright holder for this preprint (which was not certified by peer review) is the author/funder. This article is a US Government work. It is not subject to copyright under 17 USC 105 and is also made available for use under a CC0 license.

bioRxiv preprint doi: https://doi.org/10.1101/2023.07.31.551097; this version posted August 19, 2025. The copyright holder for this preprint (which was not certified by peer review) is the author/funder. This article is a US Government work. It is not subject to copyright under 17 USC 105 and is also made available for use under a CC0 license.

Supplemental Figure 4: Majority of aDEGs are protein coding across cell-types and regions
Classification of aDEGs as protein coding or long non-coding RNAs (annotations associated with refdata-gex-GRCh38-2020-A) across all cell-type-region combinations reveals that the majority of aDEGs are protein coding.

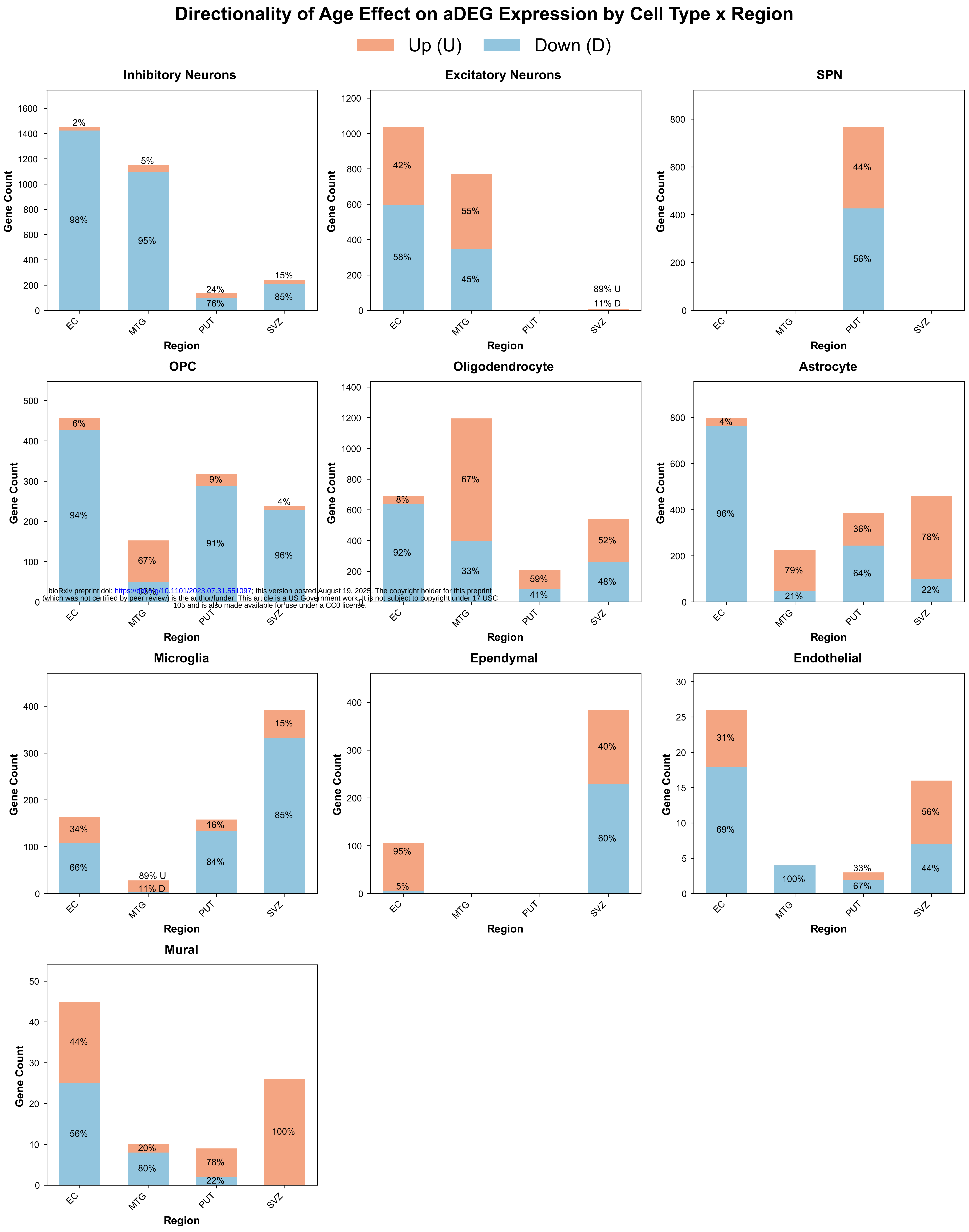

bioRxiv preprint doi: https://doi.org/10.1101/2023.07.31.551097; this version posted August 19, 2025. The copyright holder for this preprint (which was not certified by peer review) is the author/funder. This article is a US Government work. It is not subject to copyright under 17 USC 105 and is also made available for use under a CC0 license.

bioRxiv preprint doi: https://doi.org/10.1101/2023.07.31.551097; this version posted August 19, 2025. The copyright holder for this preprint (which was not certified by peer review) is the author/funder. This article is a US Government work. It is not subject to copyright under 17 USC 105 and is also made available for use under a CC0 license.

Supplemental Figure 5: aDEG expression levels tend to decrease with age across most cell-type by region combinations

aDEG expression direction (i.e. increase or decrease) across all cell-type-region combinations suggests that overall, the majority of aDEGs tend to decrease in expression with age.

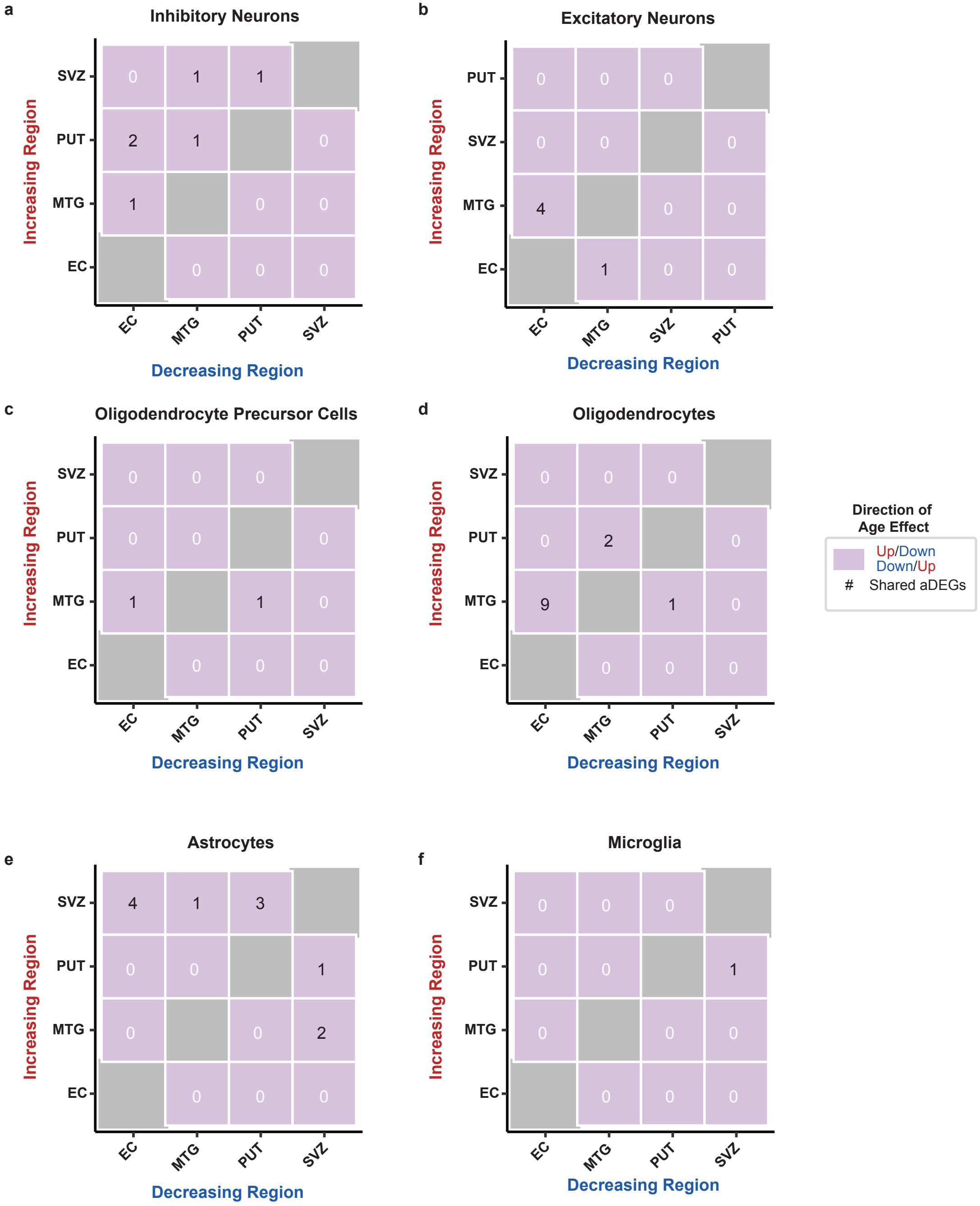
a
Inhibitory Neurons
Increasing Region
SVZ
PUT
MTG
EC
0 1 1
2 1 0
1 0 0
0 0 0
EC MTG PUT SVZ
Decreasing Region
b
Excitatory Neurons
Increasing Region
PUT
SVZ
MTG
EC
0 0 0
0 0 0
4 0 0
1 0 0
EC MTG SVZ PUT
Decreasing Region
c
Oligodendrocyte Precursor Cells
Increasing Region
SVZ
PUT
MTG
EC
0 0 0
0 0 0
1 1 0
0 0 0
EC MTG PUT SVZ
Decreasing Region
d
Oligodendrocytes
Increasing Region
SVZ
PUT
MTG
EC
0 0 0
0 2 0
9 1 0
0 0 0
EC MTG PUT SVZ
Decreasing Region
Direction of Age Effect
Up/Down
Down/Up
Shared aDEGs
e
Astrocytes
Increasing Region
SVZ
PUT
MTG
EC
4 1 3
0 0 1
0 0 2
0 0 0
EC MTG PUT SVZ
Decreasing Region
f
Microglia
Increasing Region
SVZ
PUT
MTG
EC
0 0 0
0 0 1
0 0 0
0 0 0
EC MTG PUT SVZ
Decreasing Region

bioRxiv preprint doi: https://doi.org/10.1101/2023.07.31.551097; this version posted August 19, 2025. The copyright holder for this preprint (which was not certified by peer review) is the author/funder. This article is a US Government work. It is not subject to copyright under 17 USC 105 and is also made available for use under a CC0 license.

Supplemental Figure 6: Few regionally-shared aDEGs with opposing age-effect directions across cell-types

Pairwise comparison of shared aDEGs that differ in age-effect direction between regions (discordant) within a given cell-type reveals few instances of discordant aDEGs.

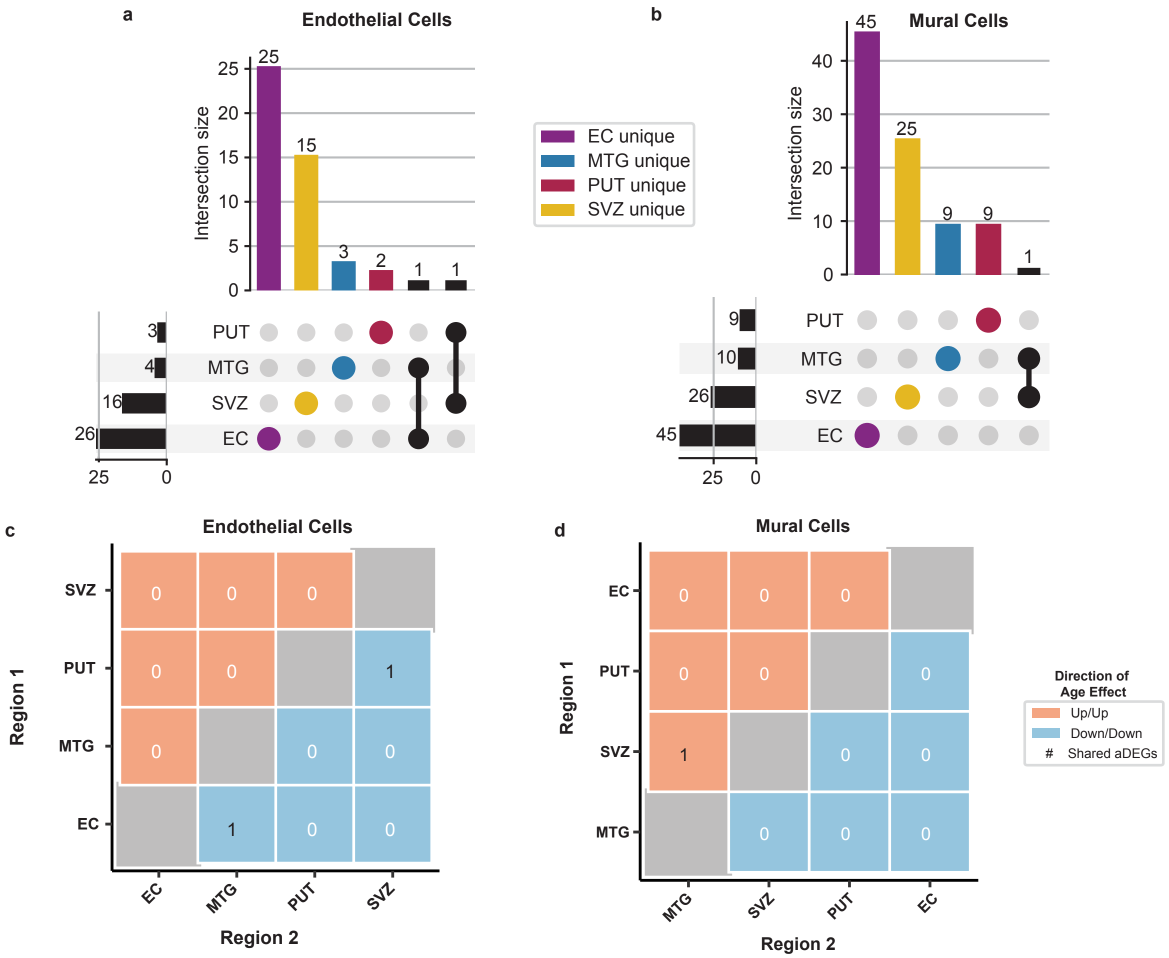

a
Endothelial Cells
Intersection size
25
15
3
2
1
1
PUT
MTG
SVZ
EC
3
4
16
26
25
0
b
Mural Cells
45
25
9
9
1
Intersection size
PUT
MTG
SVZ
EC
9
10
26
45
EC unique
MTG unique
PUT unique
SVZ unique
c
Endothelial Cells
Region 1
Region 2
SVZ
PUT
MTG
EC
d
Mural Cells
Region 1
Region 2
EC
PUT
SVZ
MTG
Direction of Age Effect
Up/Up
Down/Down
Shared aDEGs

bioRxiv preprint doi: https://doi.org/10.1101/2023.07.31.551097; this version posted August 19, 2025. The copyright holder for this preprint (which was not certified by peer review) is the author/funder. This article is a US Government work. It is not subject to copyright under 17 USC 105 and is also made available for use under a CC0 license.

Supplemental Figure 7: Pericytes exhibit few aDEGs with the majority being in the EC and SVZ.

Regional distribution and overlap of pericyte cell-type aDEGs indicate that the majority of aDEGs are unique to a particular region. Both **(a)** endothelial cells and **(b)** mural cells have the majority of aDEGs localized to the EC followed by the SVZ. Pairwise comparison of shared aDEGs within pericytes suggests little regional sharing within both cell-types. Heatmap values representing count of aDEGs indicate the number of aDEGs changing in the same direction (concordance)--either increasing (positive, red) or decreasing (negative, blue)–in both of the indicated regions. **(c)** Endothelial cells showed 2 negatively concordant aDEGs one between the EC and MTG and the other between the PUT and SVZ. **(d)** Mural cells showed 1 positively concordant aDEG between the SVZ and MTG.
