# Supplementary material for: Divergent patterns of healthy aging across human brain regions at single-cell resolution reveal links to neurodegenerative disease
Source: bioRxiv. 2023 Aug 1:2023.07.31.551097. Preprint. [Version 1] doi: 10.1101/2023.07.31.551097 (PMC10418086; doi:10.1101/2023.07.31.551097)
Supplement: Supplementary file 1 [file NIHPP2023.07.31.551097V1-supplement-1.pdf]

bioRxiv preprint doi: https://doi.org/10.1101/2023.07.31.551097; this version posted August 1, 2023. The copyright holder for this preprint (which was not certified by peer review) is the author/funder. This article is a US Government work. It is not subject to copyright under 17 USC 105 and is also made available for use under a CC0 license.

Supplemental Figure 1: Cell type composition by sex, brain region, donor and classification by broad cell type markers

(a) UMAP of 155,192 nuclei by sex (female, pink; dark blue, male)

(b) Proportion of cell types recovered by sex

(**c)** UMAP of 155,192 nuclei colored by brain region (EC, dark blue; MTG, light blue; PUT, magenta; SVZ, gold) demonstrating the region-specific origin of some cell types.

(d) Proportion of cell types recovered by region.

(e) UMAP projection of nuclei split by brain region and sample ID demonstrating no single cluster is derived from a single donor.

(f) Leiden clustering at resolution 0.85 identified 23 distinct clusters after removal of low quality or poorly distinguished clusters.

(g) Dot plot of genes used to assign broad cell type categories to Leiden clusters, where dot size indicates percentage of cells expressing a gene and color indicates level of mean expression within that cluster.

(h) Feature plots of broad cell type markers to identify clusters as broadly as neurons, excitatory neurons, cortical inhibitory neurons, spiny projection neurons, astrocytes, endothelial cells, mural cells, ependymal cells, microglia, oligodendrocyte precursor cells, and oligodendrocytes.

(i) Force-directed graph of developmental relationships between cell types.

Abbreviations: *ANPEP*, alanyl aminopeptidase, membrane; *AQP4*, aquaporin 4; *CSF1R*, colony stimulating factor 1 receptor; *DRD1*, dopamine receptor D1; *DRD2*, dopamine receptor D2; EC, entorhinal cortex; ExN, excitatory neuron; FLE, force-directed; *FLT1*, fms related receptor tyrosine kinase 1; *FOXJ1*, forkhead box J1; *GAD2*, glutamate decarboxylase 2; InN, inhibitory neuron; *MBP*, myelin basic protein; MTG, middle temporal gyrus; *OPALIN*, oligodendrocytic myelin paranodal and inner loop protein; OPC, oligodendrocyte precursor cell;

bioRxiv preprint doi: https://doi.org/10.1101/2023.07.31.551097; this version posted August 1, 2023. The copyright holder for this preprint (which was not certified by peer review) is the author/funder. This article is a US Government work. It is not subject to copyright under 17 USC 105 and is also made available for use under a CC0 license.

PDGFRA, platelet derived growth factor receptor alpha; *PLP1*, proteolipid protein 1; PUT, putamen; *RBFOX3*, RNA binding fox-1 homolog 3; *SLC17A6*, solute carrier family 17 member 6; *SLC17A7*, solute carrier family 17 member 7

Supplemental Figure 2: Unique and diverging patterns of aging across brain regions and broad cell types in absence of cell loss

(a) Percentage of nuclei recovered per brain region by age group (light blue, young; dark blue, old) with raw numbers expressed as ‘young | old’.

(b) Proportion of nuclei recovered by age group and donor showed no significant differences between age groups. n.s. = not significant; p>0.05, Welch's t-test.

(c) UpSet plot of unique vs. shared aDAGs across brain regions where rows on the x-axis correspond to [brain region] aDAG sets, columns correspond to intersection size between sets, and bar color corresponds to direction of association (blue, negative association with age; magenta, positive association with age). Lines between sets indicate shared aDAGs. Cell type sets ordered by cardinality.

(d) Clustermap of the top 5 aDAGs across brain regions (blue, negative association; magenta, positive association). Non-coding and pseudogenes distinguished by gray italics. Cell types ordered by hierarchical clustering.

(e) UpSet plot of unique and shared aDAGs across broad cell types. Only intersection sizes of ≥50 are displayed. Cell type sets ordered by cardinality.

(f) Clustermap of the top 5 positive and negative aDAGs across broad cell types. Cell types ordered by hierarchical clustering.

bioRxiv preprint doi: https://doi.org/10.1101/2023.07.31.551097; this version posted August 1, 2023. The copyright holder for this preprint (which was not certified by peer review) is the author/funder. This article is a US Government work. It is not subject to copyright under 17 USC 105 and is also made available for use under a CC0 license.

Abbreviations: aDAG, aging-differentially associated gene; EC, entorhinal cortex; ExN, excitatory neuron; InN, inhibitory neuron; MTG, middle temporal gyrus; OPC, oligodendrocyte precursor cell; PUT, putamen; SPN, spiny projection neuron; SVZ, subventricular zone

Supplemental Figure 3: Top aDAGs across cortical and subcortical InN subtypes and overlap with aging and disease gene sets

(a) Clustermap of the top 5 positive and negative aDAGs across cortical and subcortical InN subtypes (blue, negative association with age; magenta, positive association with age). Non-coding and pseudogenes distinguished by gray italics. Cell types ordered by hierarchical clustering.

(b) Enrichment of cortical and **(c)** subcortical InN subtype aDAGS from previous aging and disease perturbation gene sets from GEO.

Abbreviations: aDAG, aging-differentially associated gene; GEO, Gene Expression Omnibus; InN, inhibitory neuron; SPN, spiny projection neuron

Supplemental Figure 4: Top aDAGs across ExN subtypes and overlap with aging and disease gene sets

(a) Clustermap of the top 5 positive and negative aDAGs across ExN subtypes (blue, negative association with age; magenta, positive association with age). Non-coding and pseudogenes are distinguished by gray italics. Cell types ordered by hierarchical clustering.

bioRxiv preprint doi: https://doi.org/10.1101/2023.07.31.551097; this version posted August 1, 2023. The copyright holder for this preprint (which was not certified by peer review) is the author/funder. This article is a US Government work. It is not subject to copyright under 17 USC 105 and is also made available for use under a CC0 license.

(b) Feature scatter increased expression and association of *RHBDL3*; **(c)** decreased expression and association of *STAC*; and **(d)** increased expression and association of *STAC2* with age in ExN.

(e) Enrichment of ExN subtype aDAGS from previous aging and disease perturbation gene sets from GEO.

Abbreviations: aDAG, aging-differentially associated gene; ExN, excitatory neuron; GEO, Gene Expression Omnibus; *RHBDL3*, rhomboid like 3; *STAC*, SH3 and cysteine rich domain; *STAC2,* SH3 and cysteine rich domain 2

Supplemental Figure 5: Top aDAGs across oligodendrocyte subtypes, myelination-related genes, and overlap with aging and disease gene sets

(a) Clustermap of the top 5 positive and negative aDAGs across OPCs and oligodendrocyte subtypes (blue, negative association with age; magenta, positive association with age). Non-coding and pseudogenes are distinguished by gray italics. Cell types ordered by hierarchical clustering.

(b) Clustermap of aDAGs related to CNS myelination across cell types (blue, negative association with age; magenta, positive association with age). OPCs and oligodendrocytes are demarcated by black boxes.

(c) Enrichment of OPC and oligodendrocyte subtype aDAGS from previous aging and disease perturbation gene sets from GEO.

Abbreviations: CNS, central nervous system; ExN, excitatory neuron; GEO, Gene Expression Omnibus; OPC, oligodendrocyte precursor cell.

bioRxiv preprint doi: https://doi.org/10.1101/2023.07.31.551097; this version posted August 1, 2023. The copyright holder for this preprint (which was not certified by peer review) is the author/funder. This article is a US Government work. It is not subject to copyright under 17 USC 105 and is also made available for use under a CC0 license.

Supplemental Figure 6: Top aDAGs across non-neuronal, non-oligodendrocyte cells and overlap with aging and disease gene sets

(a) Clustermap of the top 5 positive and negative aDAGs across astrocytes, endothelial cells, mural cells, ependymal cells, and microglia (blue, negative association with age; magenta, positive association with age). Non-coding and pseudogenes are distinguished by gray italics. Cell types ordered by hierarchical clustering.

(b) UpSet plot of unique vs. shared aDAGs across astrocytes, endothelial cells, mural cells, ependymal cells, and microglia, where rows on the x-axis correspond to [cell type] aDAG sets, columns correspond to intersection size between sets, and bar color corresponds to the direction of association (blue, negative association with age; magenta, positive association with age)

(c) GSE of aDAGs in endothelial, ependymal, and mural cells.

Abbreviations: aDAG, aging-differentially associated gene; GSE, gene set enrichment

Supplemental Figure 7: Overlap of aDAGs across cell types with genes differentially expressed in aging human frontal cortex from GEO

(a) Astrocytes, microglia, and oligodendrocyte-3 aDAGs were significantly enriched for genes previously demonstrated to be increased in aged (28 years vs. 100 years) human frontal cortex (magenta, positive association; dark blue, negative association). Non-coding and pseudogenes are demarcated by gray italics. Cell types ordered by hierarchical clustering.

Asterisks mark cell types with significant enrichment.

bioRxiv preprint doi: https://doi.org/10.1101/2023.07.31.551097; this version posted August 1, 2023. The copyright holder for this preprint (which was not certified by peer review) is the author/funder. This article is a US Government work. It is not subject to copyright under 17 USC 105 and is also made available for use under a CC0 license.

(b) Feature scatter showing increased expression and association of *SERPINA3* and **(c)** *IFITM2* in with age in astrocytes

(d) Feature scatter showing increased expression and association of *CD163* and **(e)** *CD14* in with age in microglia

(f) Only neuronal subtypes were significantly enriched for genes previously demonstrated to be decreased in aged (28 years vs. 100 years) human frontal cortex (magenta, positive association; dark blue, negative association). Non-coding and pseudogenes are demarcated by gray italics. Asterisks mark cell types with significant enrichment. Cell types ordered by hierarchical clustering.

(g) Feature scatter showing decreased expression and association with age of *KCNF1* in *LHX*$^+$*PVALB*$^+$ cortical InN, **(h)** *LY6H* in cortical InN and *RORB*$^+$ ExN, and **(i)** *PFN1* in *LHX6*$^+$*PVALB*$^+$ cortical InN

Abbreviations: aDAG, aging-differentially associated gene; EC, entorhinal cortex; ExN, excitatory neuron; *IFITM2*, interferon-induced transmembrane protein 2; InN, inhibitory neuron; *KCNF1*, potassium voltage-gated channel modifier subfamily F member 1; *LY6H*, lymphocyte antigen 6 family member H; MTG, middle temporal gyrus; OPC, oligodendrocyte precursor cell; *PFN1*, profilin 1; PUT, putamen; *SERPINA3*, serpin family A member 3; SPN, spiny projection neuron; SVZ, subventricular zone; UMAP, uniform manifold approximation and projection.

bioRxiv preprint doi: https://doi.org/10.1101/2023.07.31.551097; this version posted August 1, 2023. The copyright holder for this preprint (which was not certified by peer review) is the author/funder. This article is a US Government work. It is not subject to copyright under 17 USC 105 and is also made available for use under a CC0 license.

Supplemental Figure 8: Mean expression of genes nearest to AD and PD GWAS-identified risk loci across brain regions and cell types

(a) Dot plot of mean expression of genes nearest to AD GWAS-nominated risk loci across brain regions. Size of circle denotes percentage of cells in a brain region expressing a gene and color represents strength of expression (purple, high expression; light blue, low expression)

(b) Dot plot of mean expression of genes nearest to AD GWAS-nominated risk loci across cell types

(c) Dot plot of mean expression of genes nearest to PD GWAS-nominated risk loci across brain regions and **(d)** cell types

bioRxiv preprint doi: https://doi.org/10.1101/2023.07.31.551097; this version posted August 1, 2023. The copyright holder for this preprint (which was not certified by peer review) is the author/funder. This article is a US Government work. It is not subject to copyright under 17 USC 105 and is also made available for use under a CC0 license.

Supplemental Table 1: Donor Demographics

Supplemental Table 2: Number of Significant Differentially Associated Genes with Age by Brain Region, Broad Type, and Cell Subtype

Supplemental Table 3: Coefficient estimates for significant brain region aDAGs that are also significant at the broad type level

Supplemental Table 4: glmmTMB All Significant aDAGs (FDR BH < 0.05)

Supplemental Table 5: Oligodendrocyte Proportions by Brain Region and Age

Supplemental Information: Age-Associated Gene Set Enrichment Outputs for Brain Region, Broad Type, and Cell Type

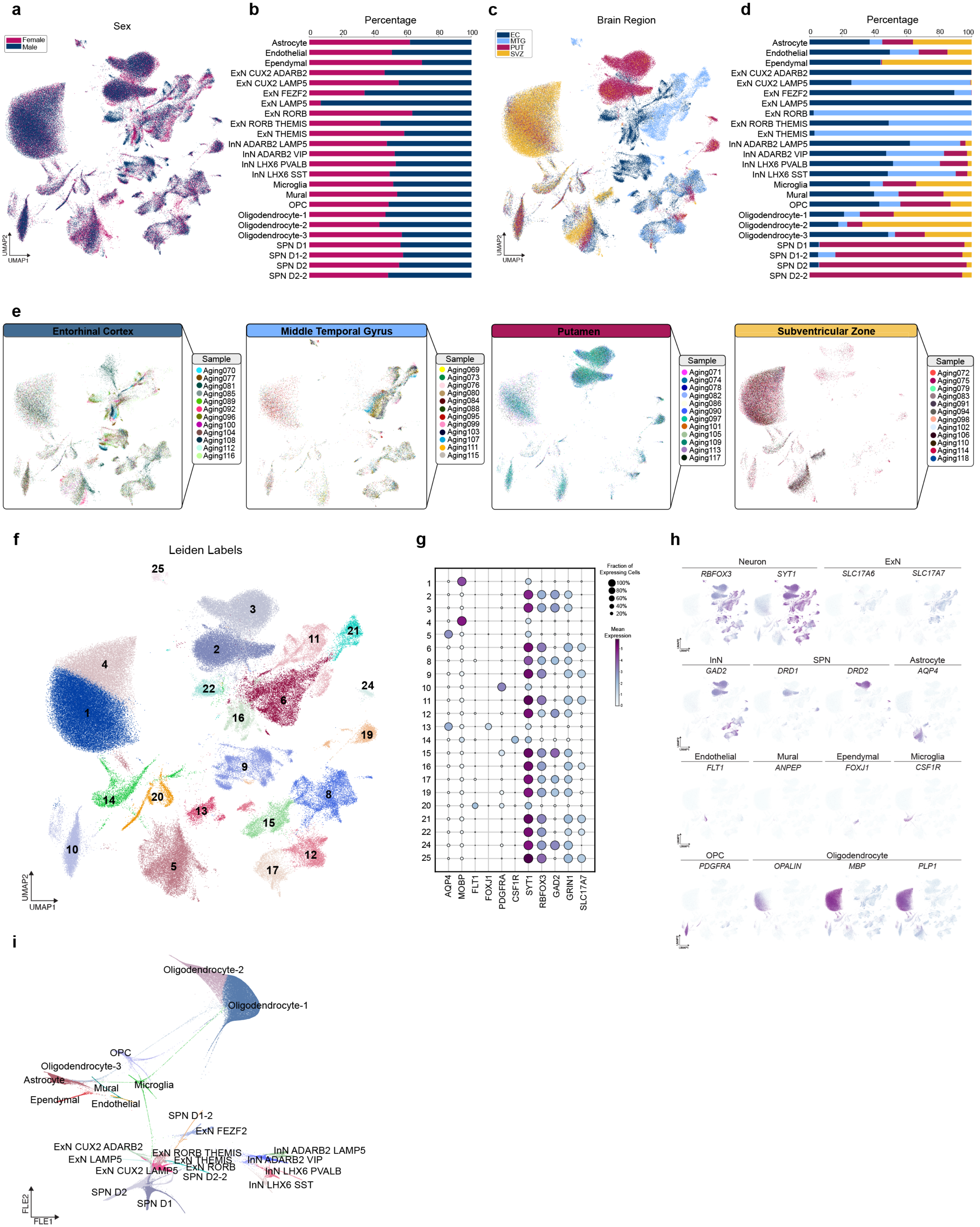
a
Sex
Female
Male
UMAP2
UMAP1
b
Percentage
0
20
40
60
80
100
Astrocyte
Endothelial
Ependymal
ExN CUX2 ADARB2
ExN CUX2 LAMP5
ExN FEZF2
ExN LAMP5
ExN RORB
ExN RORB THEMIS
ExN THEMIS
InN ADARB2 LAMP5
InN ADARB2 VIP
InN LHX6 PVALB
InN LHX6 SST
Microglia
Mural
OPC
Oligodendrocyte-1
Oligodendrocyte-2
Oligodendrocyte-3
SPN D1
SPN D1-2
SPN D2
SPN D2-2
c
Brain Region
EC
MTG
PUT
SVZ
UMAP2
UMAP1
d
Percentage
0
20
40
60
80
100
Astrocyte
Endothelial
Ependymal
ExN CUX2 ADARB2
ExN CUX2 LAMP5
ExN FEZF2
ExN LAMP5
ExN RORB
ExN RORB THEMIS
ExN THEMIS
InN ADARB2 LAMP5
InN ADARB2 VIP
InN LHX6 PVALB
InN LHX6 SST
Microglia
Mural
OPC
Oligodendrocyte-1
Oligodendrocyte-2
Oligodendrocyte-3
SPN D1
SPN D1-2
SPN D2
SPN D2-2
e
Entorhinal Cortex
Sample
Aging070
Aging077
Aging081
Aging085
Aging089
Aging092
Aging096
Aging100
Aging104
Aging108
Aging112
Aging116
Middle Temporal Gyrus
Sample
Aging069
Aging073
Aging076
Aging080
Aging084
Aging088
Aging095
Aging099
Aging103
Aging107
Aging111
Aging115
Putamen
Sample
Aging071
Aging074
Aging078
Aging082
Aging086
Aging090
Aging097
Aging101
Aging105
Aging109
Aging113
Aging117
Subventricular Zone
Sample
Aging072
Aging075
Aging079
Aging083
Aging091
Aging094
Aging098
Aging102
Aging106
Aging110
Aging114
Aging118
f
Leiden Labels
25
3
2
11
21
4
22
24
1
6
16
19
9
14
20
13
8
15
10
5
12
17
UMAP2
UMAP1
g
Fraction of Expressing Cells
100%
80%
60%
40%
20%
Mean Expression
1
2
3
4
5
6
8
9
10
11
12
13
14
15
16
17
19
20
21
22
24
25
AQP4
MOBP
FLT1
FOXJ1
PDGFRA
CSF1R
SYT1
RBFOX3
GAD2
GRIN1
SLC17A7
h
Neuron
RBFOX3
SYT1
ExN
SLC17A6
SLC17A7
InN
GAD2
SPN
DRD1
DRD2
Astrocyte
AQP4
Endothelial
FLT1
Mural
ANPEP
Ependymal
FOXJ1
Microglia
CSF1R
OPC
PDGFRA
Oligodendrocyte
OPALIN
MBP
PLP1
i
Oligodendrocyte-2
Oligodendrocyte-1
OPC
Oligodendrocyte-3
Astrocyte
Mural
Microglia
Ependymal
Endothelial
SPN D1-2
ExN FEZF2
ExN CUX2 ADARB2
ExN RORB THEMIS
InN ADARB2 LAMP5
ExN LAMP5
ExN THEMIS
InN ADARB2 VIP
ExN CUX2 LAMP5
ExN RORB
InN LHX6 PVALB
SPN D2-2
InN LHX6 SST
SPN D2
SPN D1
FLE2
FLE1

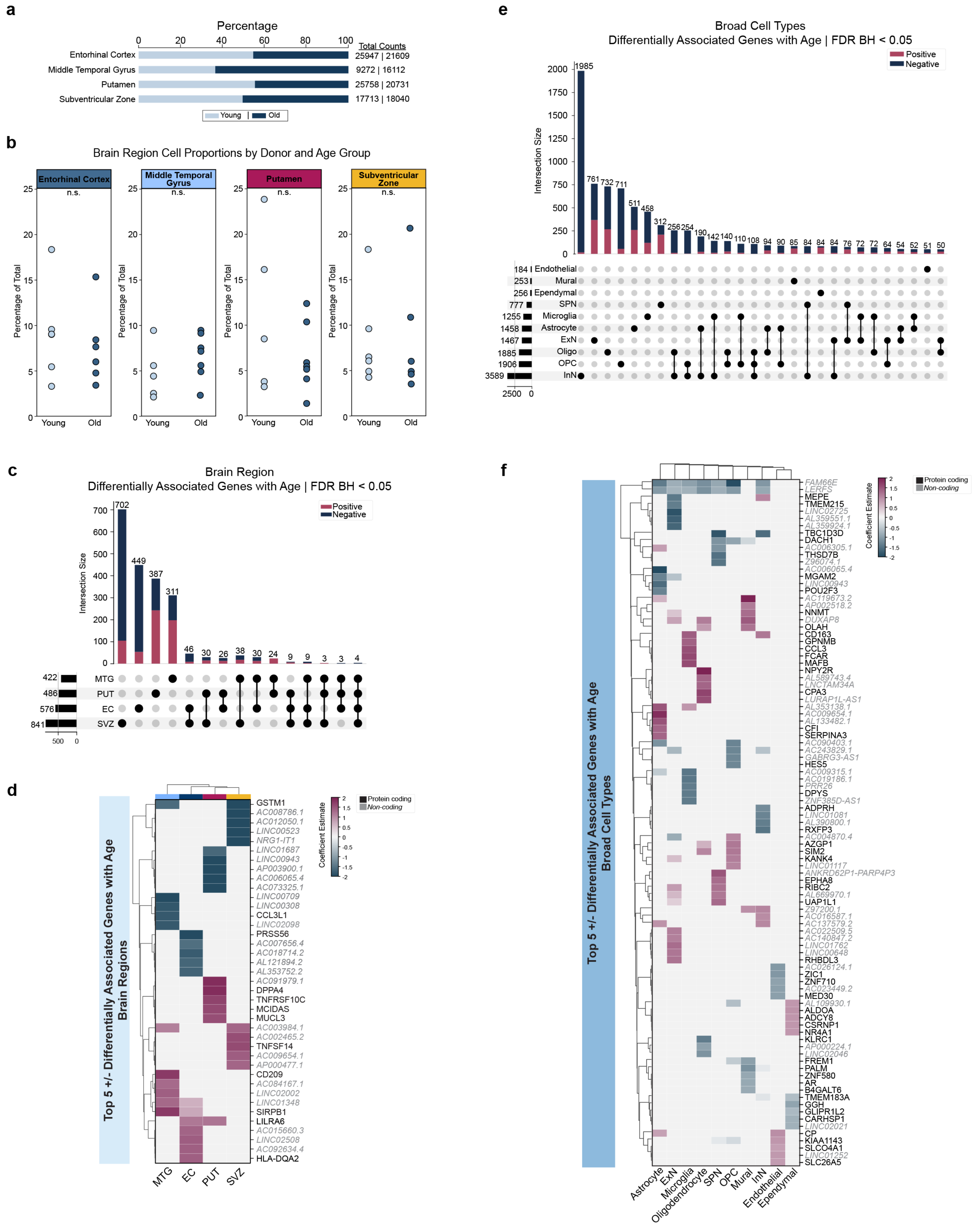

a
Percentage
Total Counts
Entorhinal Cortex 25947 | 21609
Middle Temporal Gyrus 9272 | 16112
Putamen 25758 | 20731
Subventricular Zone 17713 | 18040
Young | Old
b
Brain Region Cell Proportions by Donor and Age Group
Entorhinal Cortex
Middle Temporal Gyrus
Putamen
Subventricular Zone
n.s.
Percentage of Total
Young
Old
c
Brain Region
Differentially Associated Genes with Age | FDR BH < 0.05
Positive
Negative
Intersection Size
702 449 387 311 46 30 26 38 30 24 9 9 3 3 4
422 MTG
486 PUT
576 EC
841 SVZ
d
Top 5 +/- Differentially Associated Genes with Age
Brain Regions
Protein coding
Non-coding
Coefficient Estimate
GSTM1
AC008786.1
AC012050.1
LINC00523
NRG1-IT1
LINC01687
LINC00943
AP003900.1
AC006065.4
AC073325.1
LINC00709
LINC00308
CCL3L1
LINC02098
PRSS56
AC007656.4
AC018714.2
AL121894.2
AL353752.2
AC091979.1
DPPA4
TNFRSF10C
MCIDAS
MUCL3
AC003984.1
AC002465.2
TNFSF14
AC009654.1
AP000477.1
CD209
AC084167.1
LINC02002
LINC01348
SIRPB1
LILRA6
AC015660.3
LINC02508
AC092634.4
HLA-DQA2
MTG
EC
PUT
SVZ
e
Broad Cell Types
Differentially Associated Genes with Age | FDR BH < 0.05
Positive
Negative
Intersection Size
1985 761 732 711 511 458 312 256 254 190 142 140 110 108 94 90 85 84 84 84 76 72 72 64 54 52 51 50
184 Endothelial
253 Mural
256 Ependymal
777 SPN
1255 Microglia
1458 Astrocyte
1467 ExN
1885 Oligo
1906 OPC
3589 InN
f
Top 5 +/- Differentially Associated Genes with Age
Broad Cell Types
Protein coding
Non-coding
Coefficient Estimate
FAM66E
LERFS
MEPE
TMEM215
LINC02725
AL359551.1
AL359924.1
TBC1D3D
DACH1
AC006305.1
THSD7B
Z96074.1
AC006065.4
MGAM2
LINC00943
POU2F3
AC119673.2
AP002518.2
NNMT
DUXAP8
OLAH
CD163
GPNMB
CCL3
FCAR
MAFB
NPY2R
AL589743.4
LNCTAM34A
CPA3
LURAP1L-AS1
AL353138.1
AC009654.1
AL133482.1
CFI
SERPINA3
AC090403.1
AC243829.1
GABRG3-AS1
HES5
AC009315.1
AC019186.1
PRR26
DPYS
ZNF385D-AS1
ADPRH
LINC01081
AL390800.1
RXFP3
AC004870.4
AZGP1
SIM2
KANK4
LINC01117
ANKRD62P1-PARP4P3
EPHA8
RIBC2
AL669970.1
UAP1L1
Z97200.1
AC016587.1
AC137579.2
AC022509.5
AC140847.2
LINC01762
LINC00648
RHBDL3
AC026124.1
ZIC1
ZNF710
AC023449.2
MED30
AL109930.1
ALDOA
ADCY8
CSRNP1
NR4A1
KLRC1
AP000224.1
LINC02046
FREM1
PALM
ZNF580
AR
B4GALT6
TMEM183A
GGH
GLIPR1L2
CARHSP1
LINC02021
CP
KIAA1143
SLCO4A1
LINC01252
SLC26A5
Astrocyte
ExN
Microglia
Oligodendrocyte
SPN
OPC
Mural
InN
Endothelial
Ependymal

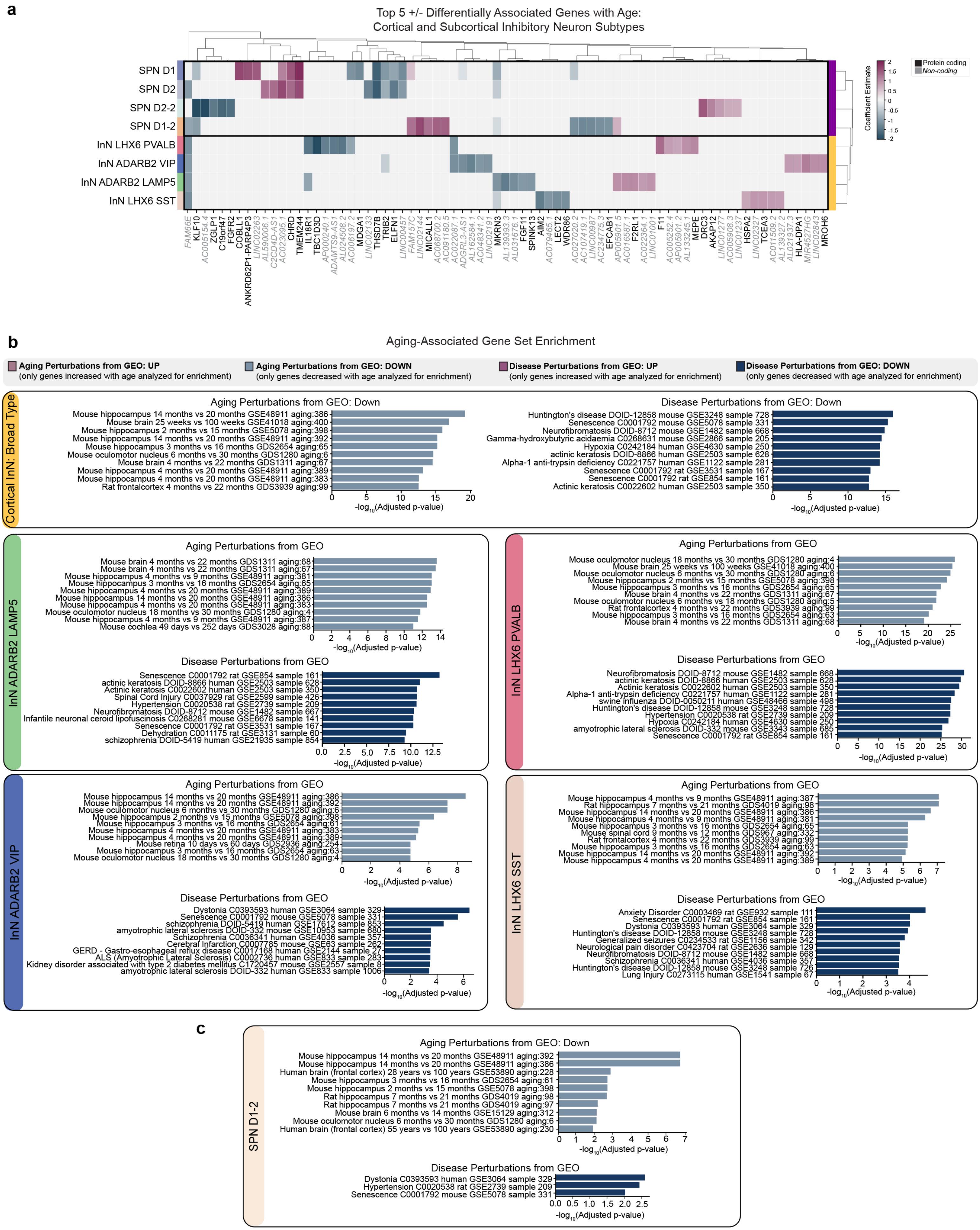
a
Top 5 +/- Differentially Associated Genes with Age:
Cortical and Subcortical Inhibitory Neuron Subtypes
SPN D1
SPN D2
SPN D2-2
SPN D1-2
InN LHX6 PVALB
InN ADARB2 VIP
InN ADARB2 LAMP5
InN LHX6 SST
Coefficient Estimate
2
1.5
1
0.5
0
-0.5
-1
-1.5
-2
Protein coding
Non-coding
FAM66E
KLF10
AC005154.4
ZGLP1
C19orf47
FGFR2
COBLL1
ANKRD62P1-PARP4P3
LINC02263
AL590006.1
C2CD4D-AS1
AC023095.1
CHRD
TMEM244
IL18R1
TBC1D3D
AP000240.1
ADAMTS9-AS1
AL024508.2
AC006197.2
MDGA1
LINC02133
THSD7B
TRIB2
ELFN1
LINC00457
FAM157C
LINC02144
MICALL1
AC068700.2
AC091180.5
AC022087.1
ADGRL3-AS1
AL162584.1
AC048341.2
LINC02191
MKRN3
AL139393.3
AL031676.1
FGF11
SPINK13
AIM2
AC079465.1
LECT2
WDR86
AC027020.2
AC107419.1
LINC00987
AC234775.3
EFCAB1
AP005901.5
AC016587.1
F2RL1
AC022364.1
LINC01001
F11
AC005252.4
AP005901.2
AL133245.1
MEPE
DRC3
AKAP12
LINC01277
AC005908.3
LINC01237
HSPA2
LINC02327
TCEA3
AC011509.2
AL139327.2
AL021937.3
HLA-DPA1
MIR4527HG
LINC02843
MROH6
b
Aging-Associated Gene Set Enrichment
Aging Perturbations from GEO: UP
(only genes increased with age analyzed for enrichment)
Aging Perturbations from GEO: DOWN
(only genes decreased with age analyzed for enrichment)
Disease Perturbations from GEO: UP
(only genes increased with age analyzed for enrichment)
Disease Perturbations from GEO: DOWN
(only genes decreased with age analyzed for enrichment)
Cortical InN: Broad Type
Aging Perturbations from GEO: Down
Mouse hippocampus 14 months vs 20 months GSE48911 aging:386
Mouse brain 25 weeks vs 100 weeks GSE41018 aging:400
Mouse hippocampus 2 months vs 15 months GSE5078 aging:398
Mouse hippocampus 14 months vs 20 months GSE48911 aging:392
Mouse hippocampus 3 months vs 16 months GDS2654 aging:65
Mouse oculomotor nucleus 6 months vs 30 months GDS1280 aging:6
Mouse brain 4 months vs 22 months GDS1311 aging:67
Mouse hippocampus 4 months vs 20 months GSE48911 aging:389
Mouse hippocampus 4 months vs 20 months GSE48911 aging:383
Rat frontalcortex 4 months vs 22 months GDS3939 aging:99
0 5 10 15 20
-log10(Adjusted p-value)
Disease Perturbations from GEO: Down
Huntington's disease DOID-12858 mouse GSE3248 sample 728
Senescence C0001792 mouse GSE5078 sample 331
Neurofibromatosis DOID-8712 mouse GSE1482 sample 668
Gamma-hydroxybutyric acidaemia C0268631 mouse GSE2866 sample 205
Hypoxia C0242184 human GSE4630 sample 250
actinic keratosis DOID-8866 human GSE2503 sample 628
Alpha-1 anti-trypsin deficiency C0221757 human GSE1122 sample 281
Senescence C0001792 rat GSE3531 sample 167
Senescence C0001792 rat GSE854 sample 161
Actinic keratosis C0022602 human GSE2503 sample 350
0 5 10 15
-log10(Adjusted p-value)
InN ADARB2 LAMP5
Aging Perturbations from GEO
Mouse brain 4 months vs 22 months GDS1311 aging:68
Mouse brain 4 months vs 22 months GDS1311 aging:67
Mouse hippocampus 4 months vs 9 months GSE48911 aging:381
Mouse hippocampus 3 months vs 16 months GDS2654 aging:65
Mouse hippocampus 4 months vs 20 months GSE48911 aging:389
Mouse hippocampus 14 months vs 20 months GSE48911 aging:386
Mouse hippocampus 4 months vs 20 months GSE48911 aging:383
Mouse oculomotor nucleus 18 months vs 30 months GDS1280 aging:4
Mouse hippocampus 4 months vs 9 months GSE48911 aging:387
Mouse cochlea 49 days vs 252 days GDS3028 aging:88
0 2 4 6 8 10 12 14
-log10(Adjusted p-value)
Disease Perturbations from GEO
Senescence C0001792 rat GSE854 sample 161
actinic keratosis DOID-8866 human GSE2503 sample 628
Actinic keratosis C0022602 human GSE2503 sample 350
Spinal Cord Injury C0037929 rat GSE2599 sample 426
Hypertension C0020538 rat GSE2739 sample 209
Neurofibromatosis DOID-8712 mouse GSE1482 sample 667
Infantile neuronal ceroid lipofuscinosis C0268281 mouse GSE6678 sample 141
Senescence C0001792 rat GSE3531 sample 167
Dehydration C0011175 rat GSE3131 sample 60
schizophrenia DOID-5419 human GSE21935 sample 854
0.0 2.5 5.0 7.5 10.0 12.5
-log10(Adjusted p-value)
InN LHX6 PVALB
Aging Perturbations from GEO
Mouse oculomotor nucleus 18 months vs 30 months GDS1280 aging:4
Mouse brain 25 weeks vs 100 weeks GSE41018 aging:400
Mouse oculomotor nucleus 6 months vs 30 months GDS1280 aging:6
Mouse hippocampus 2 months vs 15 months GSE5078 aging:398
Mouse hippocampus 3 months vs 16 months GDS2654 aging:65
Mouse brain 4 months vs 22 months GDS1311 aging:67
Mouse oculomotor nucleus 6 months vs 18 months GDS1280 aging:5
Rat frontalcortex 4 months vs 22 months GDS3939 aging:99
Mouse hippocampus 3 months vs 16 months GDS2654 aging:63
Mouse brain 4 months vs 22 months GDS1311 aging:68
0 5 10 15 20 25
-log10(Adjusted p-value)
Disease Perturbations from GEO
Neurofibromatosis DOID-8712 mouse GSE1482 sample 668
actinic keratosis DOID-8866 human GSE2503 sample 628
Actinic keratosis C0022602 human GSE2503 sample 350
Alpha-1 anti-trypsin deficiency C0221757 human GSE1122 sample 281
swine influenza DOID-0050211 human GSE48466 sample 498
Huntington's disease DOID-12858 mouse GSE3248 sample 728
Hypertension C0020538 rat GSE2739 sample 209
Hypoxia C0242184 human GSE4630 sample 250
amyotrophic lateral sclerosis DOID-332 mouse GSE3343 sample 685
Senescence C0001792 rat GSE854 sample 161
0 5 10 15 20 25 30
-log10(Adjusted p-value)
InN ADARB2 VIP
Aging Perturbations from GEO
Mouse hippocampus 14 months vs 20 months GSE48911 aging:386
Mouse hippocampus 14 months vs 20 months GSE48911 aging:392
Mouse oculomotor nucleus 6 months vs 30 months GDS1280 aging:6
Mouse hippocampus 2 months vs 15 months GSE5078 aging:398
Mouse hippocampus 3 months vs 16 months GDS2654 aging:61
Mouse hippocampus 4 months vs 20 months GSE48911 aging:383
Mouse hippocampus 4 months vs 20 months GSE48911 aging:389
Mouse retina 10 days vs 60 days GDS2936 aging:254
Mouse hippocampus 3 months vs 16 months GDS2654 aging:63
Mouse oculomotor nucleus 18 months vs 30 months GDS1280 aging:4
0 2 4 6 8
-log10(Adjusted p-value)
Disease Perturbations from GEO
Dystonia C0393593 human GSE3064 sample 329
Senescence C0001792 mouse GSE5078 sample 331
schizophrenia DOID-5419 human GSE17612 sample 853
amyotrophic lateral sclerosis DOID-332 mouse GSE10953 sample 680
Schizophrenia C0036341 human GSE4036 sample 357
Cerebral Infarction C0007785 mouse GSE63 sample 262
GERD - Gastro-esophageal reflux disease C0017168 human GSE2144 sample 27
ALS (Amyotrophic Lateral Sclerosis) C0002736 human GSE833 sample 283
Kidney disorder associated with type 2 diabetes mellitus C1720457 mouse GSE2557 sample 8
amyotrophic lateral sclerosis DOID-332 human GSE833 sample 1006
0 2 4 6
-log10(Adjusted p-value)
InN LHX6 SST
Aging Perturbations from GEO
Mouse hippocampus 4 months vs 9 months GSE48911 aging:387
Rat hippocampus 7 months vs 21 months GDS4019 aging:98
Mouse hippocampus 14 months vs 20 months GSE48911 aging:386
Mouse hippocampus 4 months vs 9 months GSE48911 aging:381
Mouse hippocampus 3 months vs 16 months GDS2654 aging:65
Mouse spinal cord 9 months vs 12 months GDS967 aging:332
Rat frontalcortex 4 months vs 22 months GDS3939 aging:99
Mouse hippocampus 3 months vs 16 months GDS2654 aging:63
Mouse hippocampus 14 months vs 20 months GSE48911 aging:392
Mouse hippocampus 4 months vs 20 months GSE48911 aging:389
0 1 2 3 4 5 6 7
-log10(Adjusted p-value)
Disease Perturbations from GEO
Anxiety Disorder C0003469 rat GSE932 sample 111
Senescence C0001792 rat GSE854 sample 161
Dystonia C0393593 human GSE3064 sample 329
Huntington's disease DOID-12858 mouse GSE3248 sample 728
Generalized seizures C0234533 rat GSE1156 sample 342
Neurological pain disorder C0423704 rat GSE2636 sample 129
Neurofibromatosis DOID-8712 mouse GSE1482 sample 668
Schizophrenia C0036341 human GSE4036 sample 357
Huntington's disease DOID-12858 mouse GSE3248 sample 726
Lung Injury C0273115 human GSE1541 sample 67
0 1 2 3 4
-log10(Adjusted p-value)
c
SPN D1-2
Aging Perturbations from GEO: Down
Mouse hippocampus 14 months vs 20 months GSE48911 aging:392
Mouse hippocampus 14 months vs 20 months GSE48911 aging:386
Human brain (frontal cortex) 28 years vs 100 years GSE53890 aging:228
Mouse hippocampus 3 months vs 16 months GDS2654 aging:61
Mouse hippocampus 2 months vs 15 months GSE5078 aging:398
Rat hippocampus 7 months vs 21 months GDS4019 aging:98
Rat hippocampus 7 months vs 21 months GDS4019 aging:97
Mouse brain 6 months vs 14 months GSE15129 aging:312
Mouse oculomotor nucleus 6 months vs 30 months GDS1280 aging:6
Human brain (frontal cortex) 55 years vs 100 years GSE53890 aging:230
0 1 2 3 4 5 6 7
-log10(Adjusted p-value)
Disease Perturbations from GEO
Dystonia C0393593 human GSE3064 sample 329
Hypertension C0020538 rat GSE2739 sample 209
Senescence C0001792 mouse GSE5078 sample 331
0.0 0.5 1.0 1.5 2.0 2.5
-log10(Adjusted p-value)

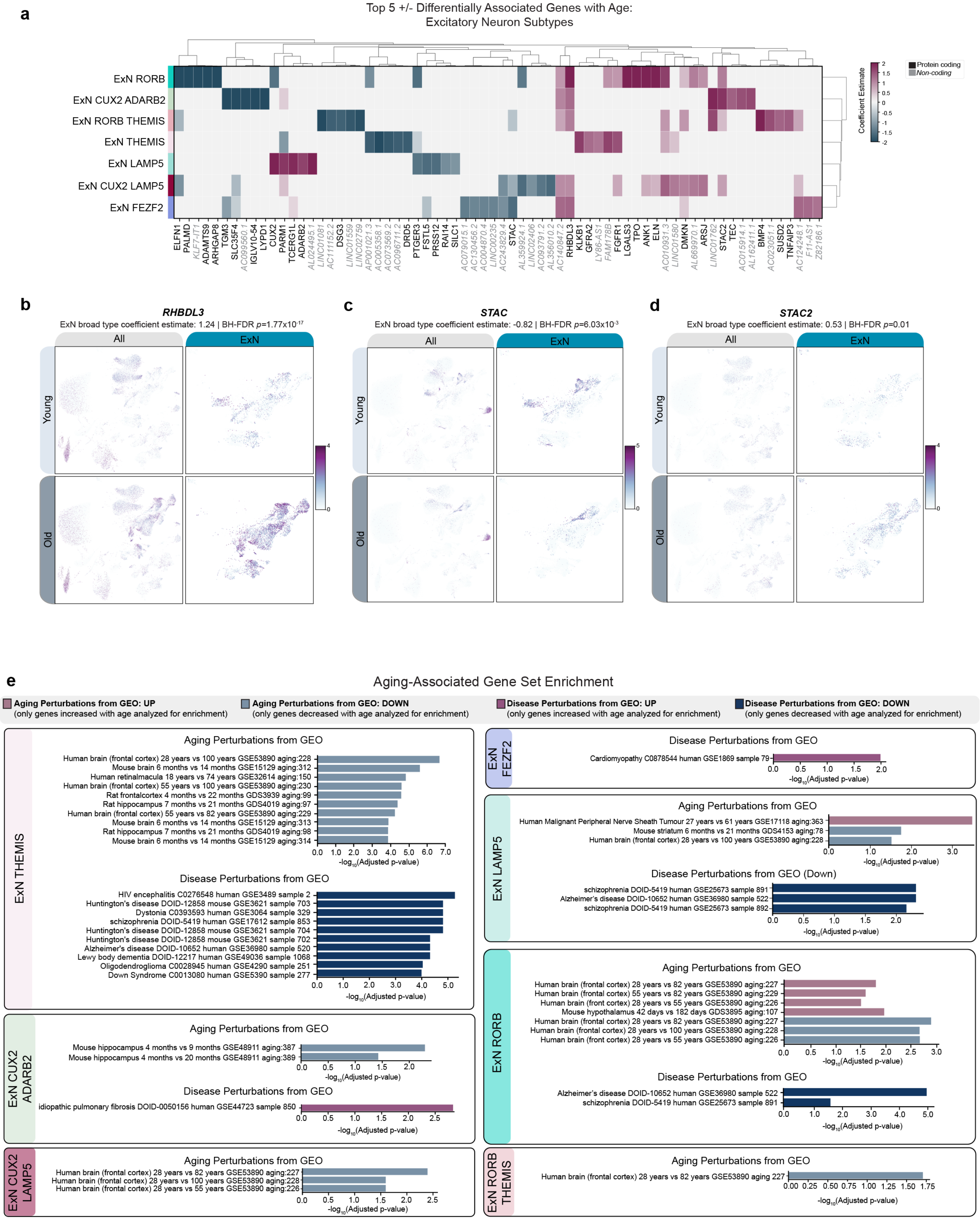
a
Top 5 +/- Differentially Associated Genes with Age:
Excitatory Neuron Subtypes
ExN RORB
ExN CUX2 ADARB2
ExN RORB THEMIS
ExN THEMIS
ExN LAMP5
ExN CUX2 LAMP5
ExN FEZF2
Coefficient Estimate
Protein coding
Non-coding
ELFN1
PALMD
KLF7-IT1
ADAMTS9
ARHGAP8
TGM3
SLC35F4
AC099560.1
IGLV10-54
LYPD1
CUX2
PARM1
TCERG1L
ADARB2
AL024495.1
LINC01081
AC111152.2
DSG3
LINC01559
LINC02759
AP001021.3
AC005358.1
AC073569.2
AC096711.2
DRD5
PTGER3
FSTL5
PRSS12
RAI14
SILC1
AC079015.1
AC130456.2
AC004870.4
LINC00235
AC243829.4
STAC
AL359924.1
LINC02406
AC093791.2
AL356010.2
AC140847.2
RHBDL3
KLKB1
GFRA2
LY86-AS1
FAM178B
FGFR1
LGALS3
TPO
ANK1
ELN
AC010931.3
LINC01580
DMKN
AL669970.1
ARSJ
LINC01762
STAC2
TEC
AC015914.1
AL162411.1
BMP4
AC023051.1
SUSD2
TNFAIP3
AC124248.1
F11-AS1
Z82186.1
b
RHBDL3
ExN broad type coefficient estimate: 1.24 | BH-FDR p=1.77x10-17
c
STAC
ExN broad type coefficient estimate: -0.82 | BH-FDR p=6.03x10-3
d
STAC2
ExN broad type coefficient estimate: 0.53 | BH-FDR p=0.01
All
ExN
Young
Old
e
Aging-Associated Gene Set Enrichment
Aging Perturbations from GEO: UP (only genes increased with age analyzed for enrichment)
Aging Perturbations from GEO: DOWN (only genes decreased with age analyzed for enrichment)
Disease Perturbations from GEO: UP (only genes increased with age analyzed for enrichment)
Disease Perturbations from GEO: DOWN (only genes decreased with age analyzed for enrichment)
ExN THEMIS
Aging Perturbations from GEO
Human brain (frontal cortex) 28 years vs 100 years GSE53890 aging:228
Mouse brain 6 months vs 14 months GSE15129 aging:312
Human retinalmacula 18 years vs 74 years GSE32614 aging:150
Human brain (frontal cortex) 55 years vs 100 years GSE53890 aging:230
Rat frontalcortex 4 months vs 22 months GDS3939 aging:99
Rat hippocampus 7 months vs 21 months GDS4019 aging:97
Human brain (frontal cortex) 55 years vs 82 years GSE53890 aging:229
Mouse brain 6 months vs 14 months GSE15129 aging:313
Rat hippocampus 7 months vs 21 months GDS4019 aging:98
Mouse brain 6 months vs 14 months GSE15129 aging:314
-log10(Adjusted p-value)
Disease Perturbations from GEO
HIV encephalitis C0276548 human GSE3489 sample 2
Huntington's disease DOID-12858 mouse GSE3621 sample 703
Dystonia C0393593 human GSE3064 sample 329
schizophrenia DOID-5419 human GSE17612 sample 853
Huntington's disease DOID-12858 mouse GSE3621 sample 704
Huntington's disease DOID-12858 mouse GSE3621 sample 702
Alzheimer's disease DOID-10652 human GSE36980 sample 520
Lewy body dementia DOID-12217 human GSE49036 sample 1068
Oligodendroglioma C0028945 human GSE4290 sample 251
Down Syndrome C0013080 human GSE5390 sample 277
-log10(Adjusted p-value)
ExN CUX2 ADARB2
Aging Perturbations from GEO
Mouse hippocampus 4 months vs 9 months GSE48911 aging:387
Mouse hippocampus 4 months vs 20 months GSE48911 aging:389
-log10(Adjusted p-value)
Disease Perturbations from GEO
idiopathic pulmonary fibrosis DOID-0050156 human GSE44723 sample 850
-log10(Adjusted p-value)
ExN CUX2 LAMP5
Aging Perturbations from GEO
Human brain (frontal cortex) 28 years vs 82 years GSE53890 aging:227
Human brain (frontal cortex) 28 years vs 100 years GSE53890 aging:228
Human brain (frontal cortex) 28 years vs 55 years GSE53890 aging:226
-log10(Adjusted p-value)
ExN FEZF2
Disease Perturbations from GEO
Cardiomyopathy C0878544 human GSE1869 sample 79
-log10(Adjusted p-value)
ExN LAMP5
Aging Perturbations from GEO
Human Malignant Peripheral Nerve Sheath Tumour 27 years vs 61 years GSE17118 aging:363
Mouse striatum 6 months vs 21 months GDS4153 aging:78
Human brain (frontal cortex) 28 years vs 100 years GSE53890 aging:228
-log10(Adjusted p-value)
Disease Perturbations from GEO (Down)
schizophrenia DOID-5419 human GSE25673 sample 891
Alzheimer's disease DOID-10652 human GSE36980 sample 522
schizophrenia DOID-5419 human GSE25673 sample 892
-log10(Adjusted p-value)
ExN RORB
Aging Perturbations from GEO
Human brain (frontal cortex) 28 years vs 82 years GSE53890 aging:227
Human brain (frontal cortex) 55 years vs 82 years GSE53890 aging:229
Human brain (front cortex) 28 years vs 55 years GSE53890 aging:226
Mouse hypothalamus 42 days vs 182 days GDS3895 aging:107
Human brain (frontal cortex) 28 years vs 82 years GSE53890 aging:227
Human brain (frontal cortex) 28 years vs 100 years GSE53890 aging:228
Human brain (front cortex) 28 years vs 55 years GSE53890 aging:226
-log10(Adjusted p-value)
Disease Perturbations from GEO
Alzheimer's disease DOID-10652 human GSE36980 sample 522
schizophrenia DOID-5419 human GSE25673 sample 891
-log10(Adjusted p-value)
ExN RORB THEMIS
Aging Perturbations from GEO
Human brain (frontal cortex) 28 years vs 82 years GSE53890 aging 227
-log10(Adjusted p-value)

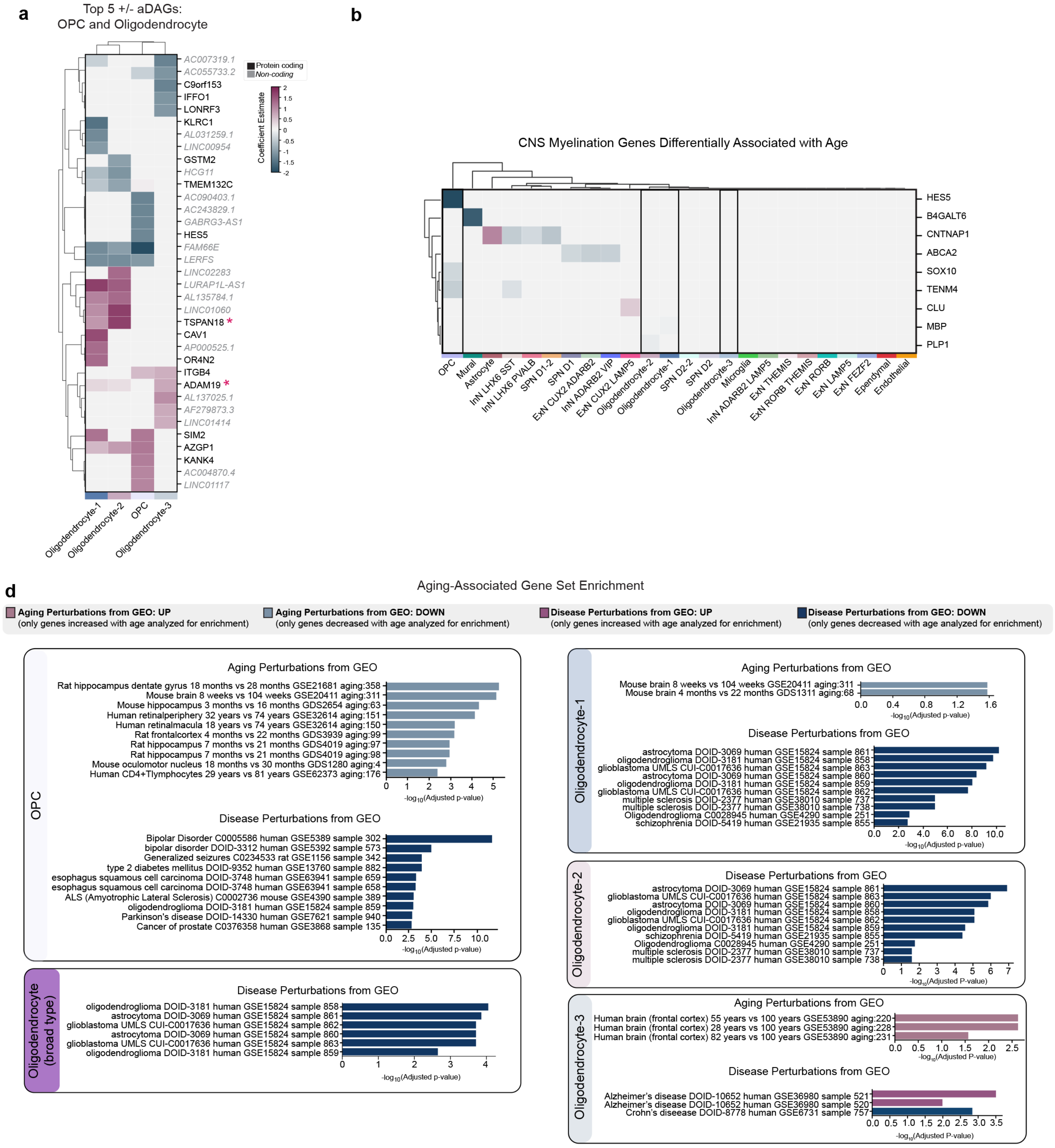

a
Top 5 +/- aDAGs:
OPC and Oligodendrocyte
Protein coding
Non-coding
Coefficient Estimate
AC007319.1
AC055733.2
C9orf153
IFFO1
LONRF3
KLRC1
AL031259.1
LINC00954
GSTM2
HCG11
TMEM132C
AC090403.1
AC243829.1
GABRG3-AS1
HES5
FAM66E
LERFS
LINC02283
LURAP1L-AS1
AL135784.1
LINC01060
TSPAN18 *
CAV1
AP000525.1
OR4N2
ITGB4
ADAM19 *
AL137025.1
AF279873.3
LINC01414
SIM2
AZGP1
KANK4
AC004870.4
LINC01117
Oligodendrocyte-1
Oligodendrocyte-2
OPC
Oligodendrocyte-3
b
CNS Myelination Genes Differentially Associated with Age
HES5
B4GALT6
CNTNAP1
ABCA2
SOX10
TENM4
CLU
MBP
PLP1
OPC
Mural
Astrocyte
InN LHX6 SST
InN LHX6 PVALB
SPN D1-2
SPN D1
ExN CUX2 ADARB2
InN ADARB2 VIP
ExN CUX2 LAMP5
Oligodendrocyte-2
Oligodendrocyte-1
SPN D2-2
SPN D2
Oligodendrocyte-3
Microglia
InN ADARB2 LAMP5
ExN THEMIS
ExN RORB THEMIS
ExN RORB
ExN LAMP5
ExN FEZF2
Ependymal
Endothelial
d
Aging-Associated Gene Set Enrichment
Aging Perturbations from GEO: UP (only genes increased with age analyzed for enrichment)
Aging Perturbations from GEO: DOWN (only genes decreased with age analyzed for enrichment)
Disease Perturbations from GEO: UP (only genes increased with age analyzed for enrichment)
Disease Perturbations from GEO: DOWN (only genes decreased with age analyzed for enrichment)
OPC
Aging Perturbations from GEO
Rat hippocampus dentate gyrus 18 months vs 28 months GSE21681 aging:358
Mouse brain 8 weeks vs 104 weeks GSE20411 aging:311
Mouse hippocampus 3 months vs 16 months GDS2654 aging:63
Human retinalperiphery 32 years vs 74 years GSE32614 aging:151
Human retinalmacula 18 years vs 74 years GSE32614 aging:150
Rat frontalcortex 4 months vs 22 months GDS3939 aging:99
Rat hippocampus 7 months vs 21 months GDS4019 aging:97
Rat hippocampus 7 months vs 21 months GDS4019 aging:98
Mouse oculomotor nucleus 18 months vs 30 months GDS1280 aging:4
Human CD4+Tlymphocytes 29 years vs 81 years GSE62373 aging:176
-log10(Adjusted p-value)
Disease Perturbations from GEO
Bipolar Disorder C0005586 human GSE5389 sample 302
bipolar disorder DOID-3312 human GSE5392 sample 573
Generalized seizures C0234533 rat GSE1156 sample 342
type 2 diabetes mellitus DOID-9352 human GSE13760 sample 882
esophagus squamous cell carcinoma DOID-3748 human GSE63941 sample 659
esophagus squamous cell carcinoma DOID-3748 human GSE63941 sample 658
ALS (Amyotrophic Lateral Sclerosis) C0002736 mouse GSE4390 sample 389
oligodendroglioma DOID-3181 human GSE15824 sample 859
Parkinson's disease DOID-14330 human GSE7621 sample 940
Cancer of prostate C0376358 human GSE3868 sample 135
-log10(Adjusted p-value)
Oligodendrocyte (broad type)
Disease Perturbations from GEO
oligodendroglioma DOID-3181 human GSE15824 sample 858
astrocytoma DOID-3069 human GSE15824 sample 861
glioblastoma UMLS CUI-C0017636 human GSE15824 sample 862
astrocytoma DOID-3069 human GSE15824 sample 860
glioblastoma UMLS CUI-C0017636 human GSE15824 sample 863
oligodendroglioma DOID-3181 human GSE15824 sample 859
-log10(Adjusted p-value)
Oligodendrocyte-1
Aging Perturbations from GEO
Mouse brain 8 weeks vs 104 weeks GSE20411 aging:311
Mouse brain 4 months vs 22 months GDS1311 aging:68
-log10(Adjusted p-value)
Disease Perturbations from GEO
astrocytoma DOID-3069 human GSE15824 sample 861
oligodendroglioma DOID-3181 human GSE15824 sample 858
glioblastoma UMLS CUI-C0017636 human GSE15824 sample 863
astrocytoma DOID-3069 human GSE15824 sample 860
oligodendroglioma DOID-3181 human GSE15824 sample 859
glioblastoma UMLS CUI-C0017636 human GSE15824 sample 862
multiple sclerosis DOID-2377 human GSE38010 sample 737
multiple sclerosis DOID-2377 human GSE38010 sample 738
Oligodendroglioma C0028945 human GSE4290 sample 251
schizophrenia DOID-5419 human GSE21935 sample 855
-log10(Adjusted p-value)
Oligodendrocyte-2
Disease Perturbations from GEO
astrocytoma DOID-3069 human GSE15824 sample 861
glioblastoma UMLS CUI-C0017636 human GSE15824 sample 863
astrocytoma DOID-3069 human GSE15824 sample 860
oligodendroglioma DOID-3181 human GSE15824 sample 858
glioblastoma UMLS CUI-C0017636 human GSE15824 sample 862
oligodendroglioma DOID-3181 human GSE15824 sample 859
schizophrenia DOID-5419 human GSE21935 sample 855
Oligodendroglioma C0028945 human GSE4290 sample 251
multiple sclerosis DOID-2377 human GSE38010 sample 737
multiple sclerosis DOID-2377 human GSE38010 sample 738
-log10(Adjusted P-value)
Oligodendrocyte-3
Aging Perturbations from GEO
Human brain (frontal cortex) 55 years vs 100 years GSE53890 aging:220
Human brain (frontal cortex) 28 years vs 100 years GSE53890 aging:228
Human brain (frontal cortex) 82 years vs 100 years GSE53890 aging:231
-log10(Adjusted P-value)
Disease Perturbations from GEO
Alzheimer's disease DOID-10652 human GSE36980 sample 521
Alzheimer's disease DOID-10652 human GSE36980 sample 520
Crohn's diseease DOID-8778 human GSE6731 sample 757
-log10(Adjusted P-value)

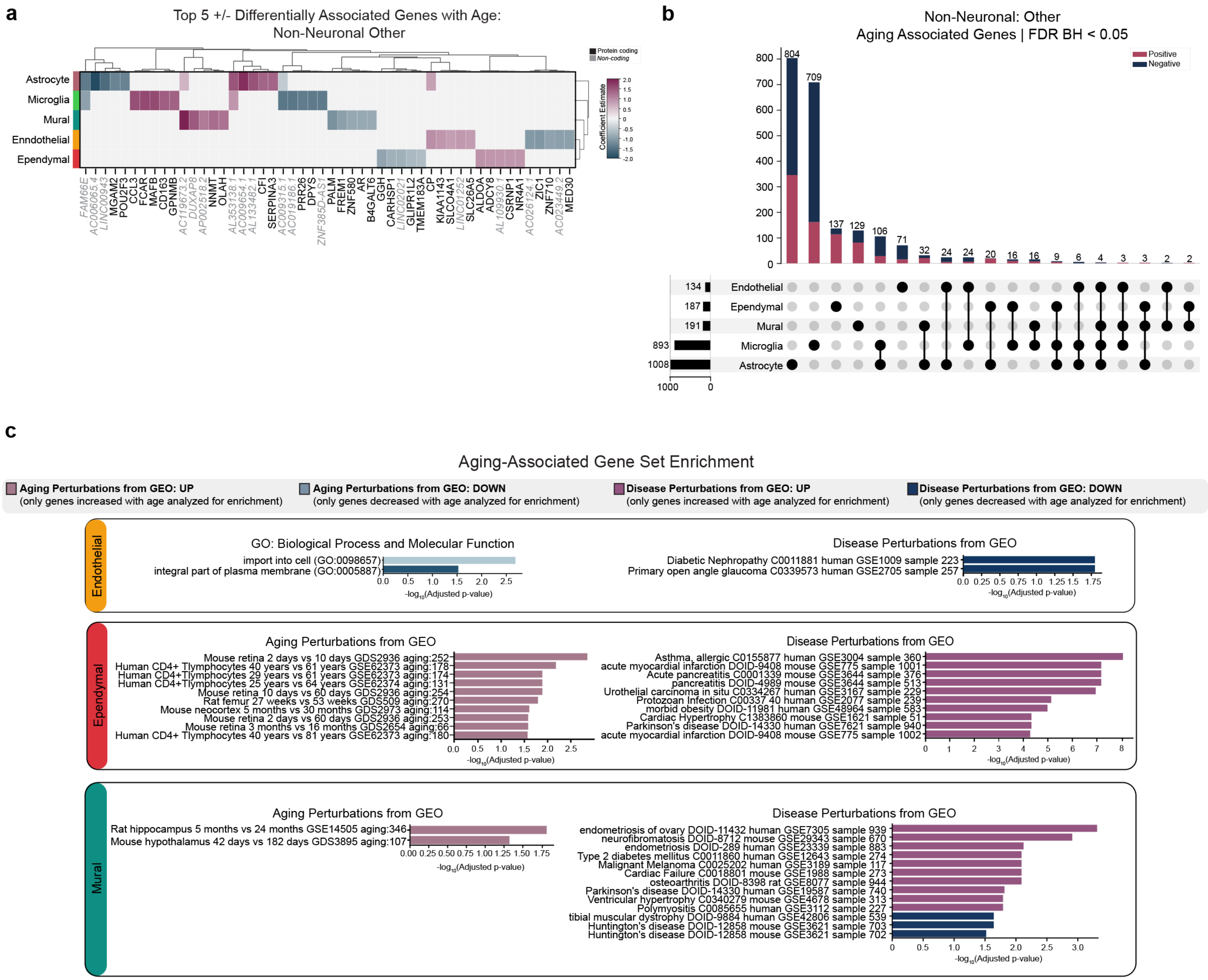

a
Top 5 +/- Differentially Associated Genes with Age:
Non-Neuronal Other
Protein coding
Non-coding
Coefficient Estimate
Astrocyte
Microglia
Mural
Enndothelial
Ependymal
FAM66E
AC006065.4
LINC00943
MGAM2
POU2F3
CCL3
FCAR
MAFB
CD163
GPNMB
AC119673.2
DUXAP8
AP002518.2
NNMT
OLAH
AL353138.1
AC009654.1
AL133482.1
CFI
SERPINA3
AC009315.1
AC019186.1
PRR26
DPYS
ZNF385D-AS1
PALM
FREM1
ZNF580
AR
B4GALT6
GGH
CARHSP1
LINC02021
GLIPR1L2
TMEM183A
CP
KIAA1143
SLCO4A1
LINC01252
SLC26A5
ALDOA
ADCY8
AL109930.1
CSRNP1
NR4A1
AC026124.1
ZIC1
ZNF710
AC023449.2
MED30
b
Non-Neuronal: Other
Aging Associated Genes | FDR BH < 0.05
Positive
Negative
804
709
137
129
106
71
32
24
24
20
16
16
9
6
4
3
3
2
2
Endothelial
Ependymal
Mural
Microglia
Astrocyte
134
187
191
893
1008
c
Aging-Associated Gene Set Enrichment
Aging Perturbations from GEO: UP
(only genes increased with age analyzed for enrichment)
Aging Perturbations from GEO: DOWN
(only genes decreased with age analyzed for enrichment)
Disease Perturbations from GEO: UP
(only genes increased with age analyzed for enrichment)
Disease Perturbations from GEO: DOWN
(only genes decreased with age analyzed for enrichment)
Endothelial
GO: Biological Process and Molecular Function
import into cell (GO:0098657)
integral part of plasma membrane (GO:0005887)
-log10(Adjusted p-value)
Disease Perturbations from GEO
Diabetic Nephropathy C0011881 human GSE1009 sample 223
Primary open angle glaucoma C0339573 human GSE2705 sample 257
-log10(Adjusted p-value)
Ependymal
Aging Perturbations from GEO
Mouse retina 2 days vs 10 days GDS2936 aging:252
Human CD4+ Tlymphocytes 40 years vs 61 years GSE62373 aging:178
Human CD4+Tlymphocytes 29 years vs 61 years GSE62373 aging:174
Human CD4+Tlymphocytes 25 years vs 64 years GSE62374 aging:131
Mouse retina 10 days vs 60 days GDS2936 aging:254
Rat femur 27 weeks vs 53 weeks GDS509 aging:270
Mouse neocortex 5 months vs 30 months GDS2973 aging:114
Mouse retina 2 days vs 60 days GDS2936 aging:253
Mouse retina 3 months vs 16 months GDS2654 aging:66
Human CD4+ Tlymphocytes 40 years vs 81 years GSE62373 aging:180
-log10(Adjusted p-value)
Disease Perturbations from GEO
Asthma, allergic C0155877 human GSE3004 sample 360
acute myocardial infarction DOID-9408 mouse GSE775 sample 1001
Acute pancreatitis C0001339 mouse GSE3644 sample 376
pancreatitis DOID-4989 mouse GSE3644 sample 513
Urothelial carcinoma in situ C0334267 human GSE3167 sample 229
Protozoan Infection C00337 40 human GSE2077 sample 239
morbid obesity DOID-11981 human GSE48964 sample 583
Cardiac Hypertrophy C1383860 mouse GSE1621 sample 51
Parkinson's disease DOID-14330 human GSE7621 sample 940
acute myocardial infarction DOID-9408 mouse GSE775 sample 1002
-log10(Adjusted p-value)
Mural
Aging Perturbations from GEO
Rat hippocampus 5 months vs 24 months GSE14505 aging:346
Mouse hypothalamus 42 days vs 182 days GDS3895 aging:107
-log10(Adjusted p-value)
Disease Perturbations from GEO
endometriosis of ovary DOID-11432 human GSE7305 sample 939
neurofibromatosis DOID-8712 mouse GSE29343 sample 670
endometriosis DOID-289 human GSE23339 sample 883
Type 2 diabetes mellitus C0011860 human GSE12643 sample 274
Malignant Melanoma C0025202 human GSE3189 sample 117
Cardiac Failure C0018801 mouse GSE1988 sample 273
osteoarthritis DOID-8398 rat GSE8077 sample 944
Parkinson's disease DOID-14330 human GSE19587 sample 740
Ventricular hypertrophy C0340279 mouse GSE4678 sample 313
Polymyositis C0085655 human GSE3112 sample 227
tibial muscular dystrophy DOID-9884 human GSE42806 sample 539
Huntington's disease DOID-12858 mouse GSE3621 sample 703
Huntington's disease DOID-12858 mouse GSE3621 sample 702
-log10(Adjusted p-value)

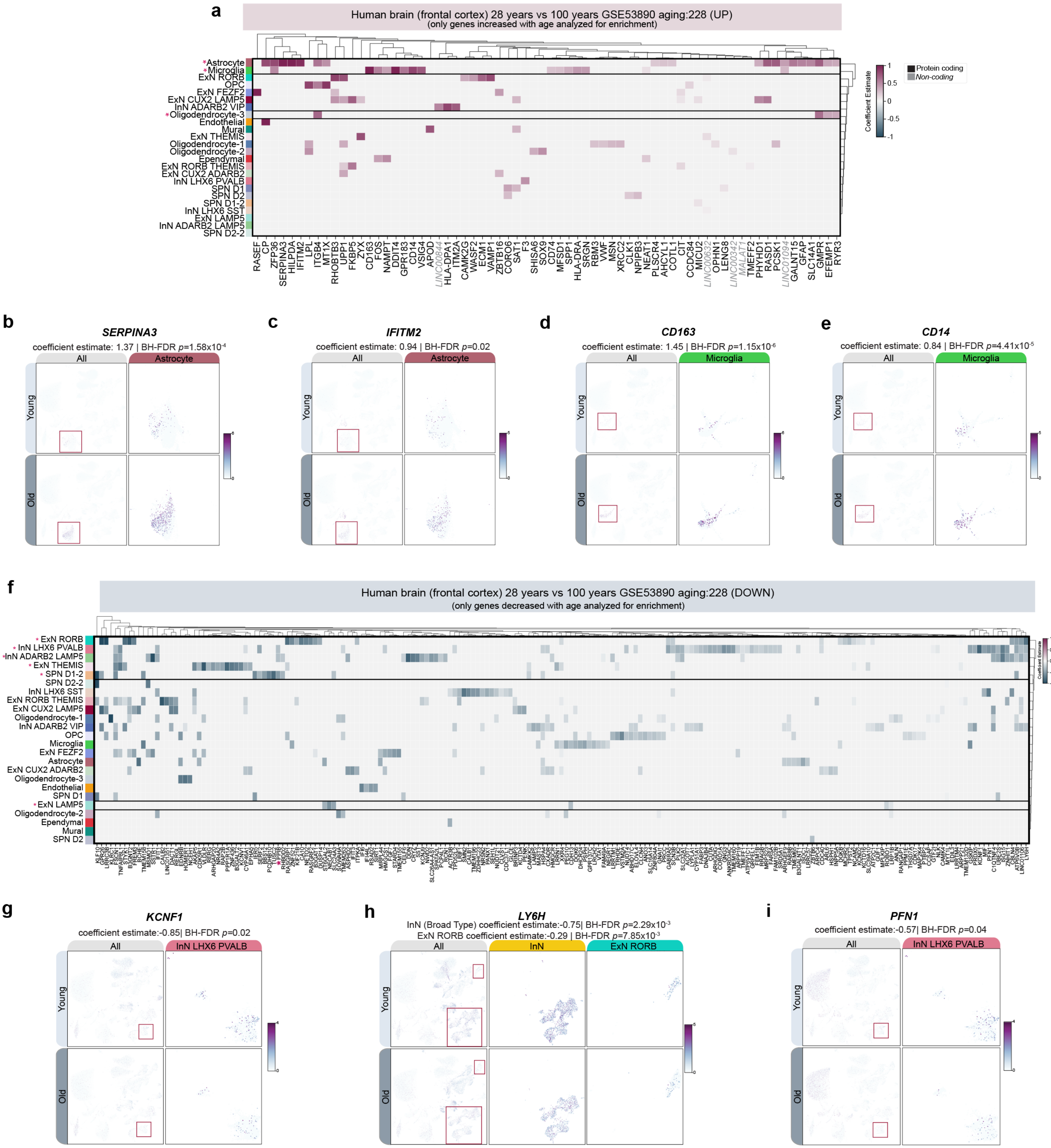
a
Human brain (frontal cortex) 28 years vs 100 years GSE53890 aging:228 (UP)
(only genes increased with age analyzed for enrichment)
*Astrocyte
*Microglia
ExN RORB
OPC
ExN FEZF2
ExN CUX2 LAMP5
InN ADARB2 VIP
*Oligodendrocyte-3
Endothelial
Mural
ExN THEMIS
Oligodendrocyte-1
Oligodendrocyte-2
Ependymal
ExN RORB THEMIS
ExN CUX2 ADARB2
InN LHX6 PVALB
SPN D1
SPN D2
SPN D1-2
InN LHX6 SST
ExN LAMP5
InN ADARB2 LAMP5
SPN D2-2
RASEF
CP
ZFP36
SERPINA3
HILPDA
IFITM2
LPL
ITGB4
MT1X
RHOBTB3
UPP1
FKBP5
ZYX
CD163
FOS
NAMPT
DDIT4
GPR183
CD14
VSIG4
APOD
LINC00844
HLA-DPA1
ITM2A
CAMK2G
WASF2
ECM1
VAMP1
ZBTB16
CORO6
SAT1
F3
SHISA6
SOX9
CD74
MFSD1
SPP1
HLA-DRA
SRGN
RBM3
VWF
MSN
XRCC2
CLK1
NPIPB3
NEAT1
PLSCR4
AHCYL1
COTL1
CIT
CCDC84
LINC00632
MICU2
OPHN1
LENG8
LINC00342
MALAT1
TMEFF2
PHYHD1
RASD1
PCSK1
LINC01094
GALNT15
GFAP
SLC14A1
GMPR
EFEMP1
RYR3
Coefficient Estimate
1
0.5
0
-0.5
-1
Protein coding
Non-coding
b
SERPINA3
coefficient estimate: 1.37 | BH-FDR p=1.58x10-4
All
Astrocyte
Young
Old
c
IFITM2
coefficient estimate: 0.94 | BH-FDR p=0.02
All
Astrocyte
Young
Old
d
CD163
coefficient estimate: 1.45 | BH-FDR p=1.15x10-6
All
Microglia
Young
Old
e
CD14
coefficient estimate: 0.84 | BH-FDR p=4.41x10-5
All
Microglia
Young
Old
f
Human brain (frontal cortex) 28 years vs 100 years GSE53890 aging:228 (DOWN)
(only genes decreased with age analyzed for enrichment)
* ExN RORB
* InN LHX6 PVALB
*InN ADARB2 LAMP5
* ExN THEMIS
* SPN D1-2
SPN D2-2
InN LHX6 SST
ExN RORB THEMIS
ExN CUX2 LAMP5
Oligodendrocyte-1
InN ADARB2 VIP
OPC
Microglia
ExN FEZF2
Astrocyte
ExN CUX2 ADARB2
Oligodendrocyte-3
Endothelial
SPN D1
* ExN LAMP5
Oligodendrocyte-2
Ependymal
Mural
SPN D2
g
KCNF1
coefficient estimate:-0.85| BH-FDR p=0.02
All
InN LHX6 PVALB
Young
Old
h
LY6H
InN (Broad Type) coefficient estimate:-0.75| BH-FDR p=2.29x10-3
ExN RORB coefficient estimate:-0.29 | BH-FDR p=7.85x10-3
All
InN
ExN RORB
Young
Old
i
PFN1
coefficient estimate:-0.57| BH-FDR p=0.04
All
InN LHX6 PVALB
Young
Old

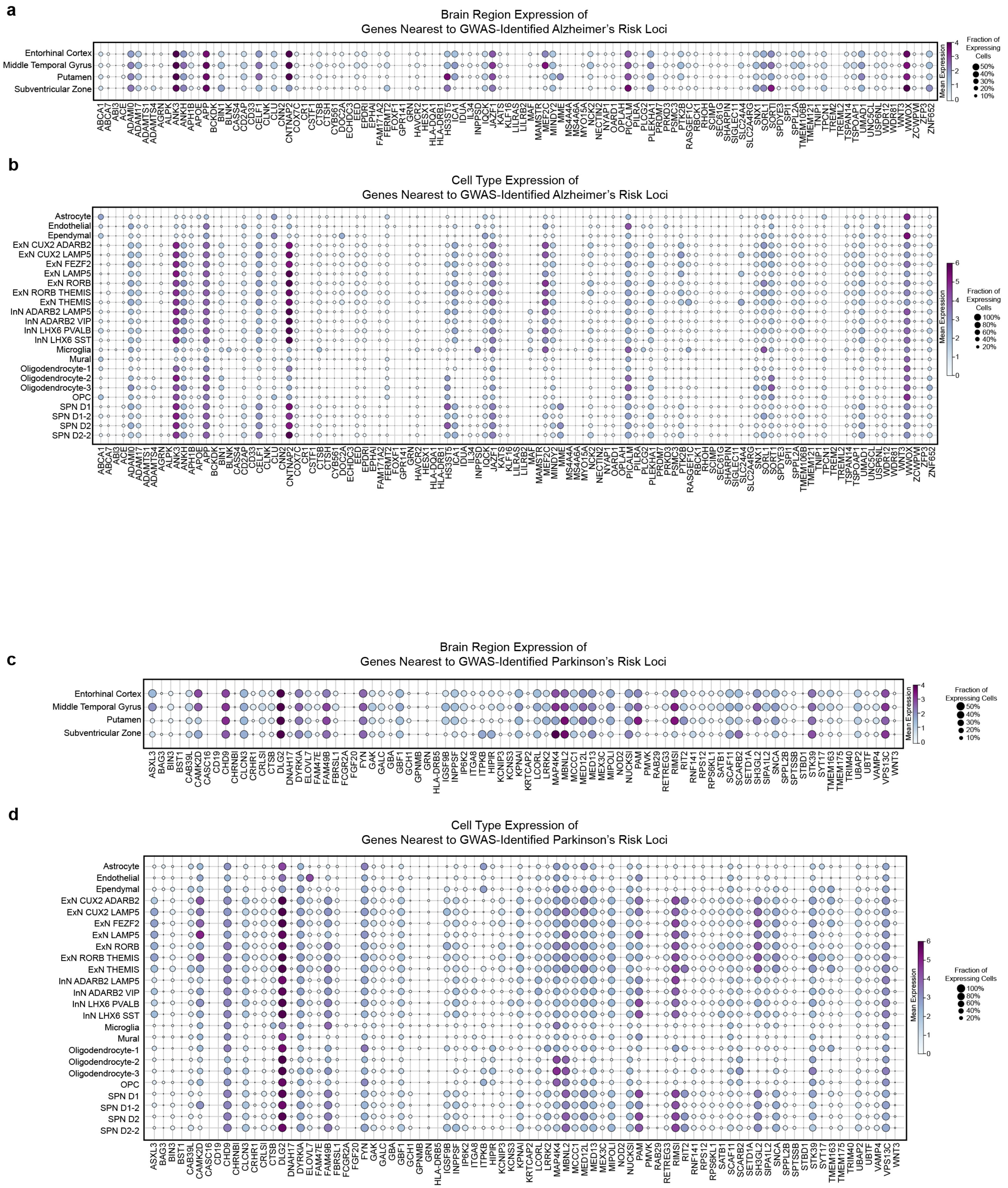

a
Brain Region Expression of Genes Nearest to GWAS-Identified Alzheimer's Risk Loci
Entorhinal Cortex
Middle Temporal Gyrus
Putamen
Subventricular Zone
Mean Expression
Fraction of Expressing Cells
50%
40%
30%
20%
10%
b
Cell Type Expression of Genes Nearest to GWAS-Identified Alzheimer's Risk Loci
Astrocyte
Endothelial
Ependymal
ExN CUX2 ADARB2
ExN CUX2 LAMP5
ExN FEZF2
ExN LAMP5
ExN RORB
ExN RORB THEMIS
ExN THEMIS
InN ADARB2 LAMP5
InN ADARB2 VIP
InN LHX6 PVALB
InN LHX6 SST
Microglia
Mural
Oligodendrocyte-1
Oligodendrocyte-2
Oligodendrocyte-3
OPC
SPN D1
SPN D1-2
SPN D2
SPN D2-2
Mean Expression
Fraction of Expressing Cells
100%
80%
60%
40%
20%
c
Brain Region Expression of Genes Nearest to GWAS-Identified Parkinson's Risk Loci
Entorhinal Cortex
Middle Temporal Gyrus
Putamen
Subventricular Zone
Mean Expression
Fraction of Expressing Cells
50%
40%
30%
20%
10%
d
Cell Type Expression of Genes Nearest to GWAS-Identified Parkinson's Risk Loci
Mean Expression
Fraction of Expressing Cells
100%
80%
60%
40%
20%
